# Interfacial Chemistry‐Tailored Silica&Metal‐Based Heterostructures: from Rational Design to Antibacterial Applications

**DOI:** 10.1002/smll.202600038

**Published:** 2026-03-24

**Authors:** Dan Cheng, Yuchao Gu, Chengzhong Yu

**Affiliations:** ^1^ College of Biological Engineering Qingdao University of Science and Technology Qingdao China; ^2^ Australian Institute for Bioengineering and Nanotechnology The University of Queensland Brisbane Australia; ^3^ Foshan (South China) Institute for New Materials Foshan China

**Keywords:** anti‐bacteria, interfacial chemistry, silica&metal‐based heterostructures

## Abstract

The escalating global crisis of antimicrobial resistance urgently demands innovative antimicrobial agents beyond conventional antibiotics. Metal‐based nanomaterials, including metal/metal oxide nanoparticles (NPs) and metal‐organic frameworks (MOFs), represent a promising class of broad‐spectrum antibacterial agents. However, their practical application is often hindered by intrinsic limitations such as aggregation, instability, and cytotoxicity. Integrating them with structurally and chemically tunable silica nanoparticles has been demonstrated as a promising strategy to mitigate the limitations and engineer advanced nanohybrids with synergistic functionalities. This review highlights how tailored interfacial chemistry achieves precise architectural control over silica&metal‐based nanohybrids. The controlled nanoarchitecture is essential to fully exploit the structural and functional contributions of silica components to overcome the physiochemical limitations of meta‐based material. We then examine the biological performance of these heterostructures mainly in antibacterial fields, including membrane disruption, stimuli‐responsive activation, biofilm penetration/eradication, and receptor‐mediated active targeting to pathogenic bacteria. Finally, challenges and future research directions are outlined based on our own perspectives, providing a design framework for next‐generation antimicrobial nanotherapeutics.

## Graphical abstract

This review explores how interfacial engineering of silica&metal‐based nanohybrids enables precise architectural control, leveraging the structural and functional contributions of silica to overcome the inherent limitations of meta‐based materials. The antibacterial performance of these nanohybrids including membrane disruption, stimuli‐responsive activation, biofilm penetration/eradication and active bacterial targeting is discussed to guide the rational design of next‐generation antimicrobial nanotherapeutics.

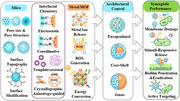

## 1 Introduction

Pathogenic bacterial infections pose a significant threat to global public health. Although antibiotics remain a cornerstone of modern medicine, their extensive and often inappropriate use has accelerated the emergence and global evolution of multidrug‐resistant bacterial strains [1, 2, 3]. The severity of this crisis is underscored by a recent estimate attributing approximately 1.27 million deaths per year to antimicrobial resistance, with projections indicating a potential rise to 10 million annually by 2050 [4]. This challenge is further exacerbated by the ability of pathogens such as methicillin‐resistant *Staphylococcus aureus* (MRSA) to form resilient biofilms. These highly organized microbial communities secrete a protective extracellular matrix that establishes a formidable physicochemical barrier, impeding antibiotic permeation, reducing bioactive drug concentration at target sites, and creating a protected microenvironment for persistent infections [5, 6]. Therefore, overcoming this dual challenge has become an urgent imperative, driving the research for innovative antibacterial strategies beyond conventional pharmacological paradigms.

Nanotechnology plays a pivotal role in antibacterial strategies via employing versatile nanocarriers, such as liposomes, polymeric, and inorganic nanoparticles. These nanoplatforms enhance therapeutic delivery by improving drug solubility, prolonging systemic circulation, and enabling controlled release [7, 8, 9]. Among them, metal‐based materials, including metal/metal oxide nanoparticles [10, 11, 12] and metal‐organic frameworks (MOFs) [13, 14, 15], have emerged as promising broad‐spectrum antibacterial agents. Their potency derives from multimodal mechanisms which can be categorized into three pathways: (1) the release of biocidal metal ions, which disrupt membrane integrity and interfere with essential cellular functions, (2) the catalytic or stimuli‐activated generation of reactive oxygen species (ROS), inducing lethal oxidative stress, and (3) the capability for localized energy conversion under external stimuli such as light and magnetic fields [16, 17]. However, their application and future clinical translation is often hindered by inherent physicochemical properties and limited structural controllability, including poor colloidal stability, severe aggregation in physiological environments, off‐target cytotoxicity toward mammalian cells, and uncontrolled release kinetics of metal ions [18, 19].

To address these challenges, silica nanomaterials have attracted considerable attention as an ideal platform for constructing functional nanohybrids [20, 21, 22]. Silica nanoparticles as carrier materials have numerous advantages, for example, tunable porosity, versatile structure, excellent biocompatibility, and abundant surface silanol groups for facile functionalization. Moreover, silica nanoparticles act not merely as passive carriers but active biomodulators [23]. For instance, the porous channel of silica can be tailored to spatially confine and stabilize metallic‐based components, preventing their aggregation and enhancing the local bioavailability as well as biological efficacy [24]. Moreover, both the pore size and particle size of silica nanoparticles can be optimized to improve the delivery efficiency of encapsulated antimicrobial enzymes [25]. In addition, engineering silica nanoparticles with rough [26] or spiky [27] surface topographies have shown enhanced interaction with bacterial membranes, facilitating the localized and sustained release of therapeutic antibacterial agents and achieving long‐term bacterial inhibition.

The progress in antibacterial silica&metal‐based nanoparticles has been summarized in several excellent reviews, typically through incorporating conventional metals (e.g., Ag, Cu, Zn) and their oxides into bioinert silica carriers [28, 29, 30]. However, their focus is largely confined to elucidating the antibacterial mechanism contributed by metal species, the contribution of silica nanoparticles is largely overlooked. Moreover, the structural design is limited to simple surface immobilization or pore encapsulation. Silica&metal‐based heterostructures incorporating advanced metal components like MOFs have also been reviewed with focus mainly on synthetic methodologies (e.g., co‐condensation, post‐grafting, supercritical CO_2_‐assisted deposition, impregnation) and broad applications in catalysis, sensing, separation, drug delivery, and bio‐imaging [31, 32, 33, 34, 35, 36]. Taking together, a systematic and dedicated review on precise structural engineering of antibacterial silica&metal‐based nanoarchitectures (including metal, metal oxide/sulfide, MOFs), modulating the multimodal antibacterial performance and understanding structure‐property relationships is still lacking.

**FIGURE 1:**
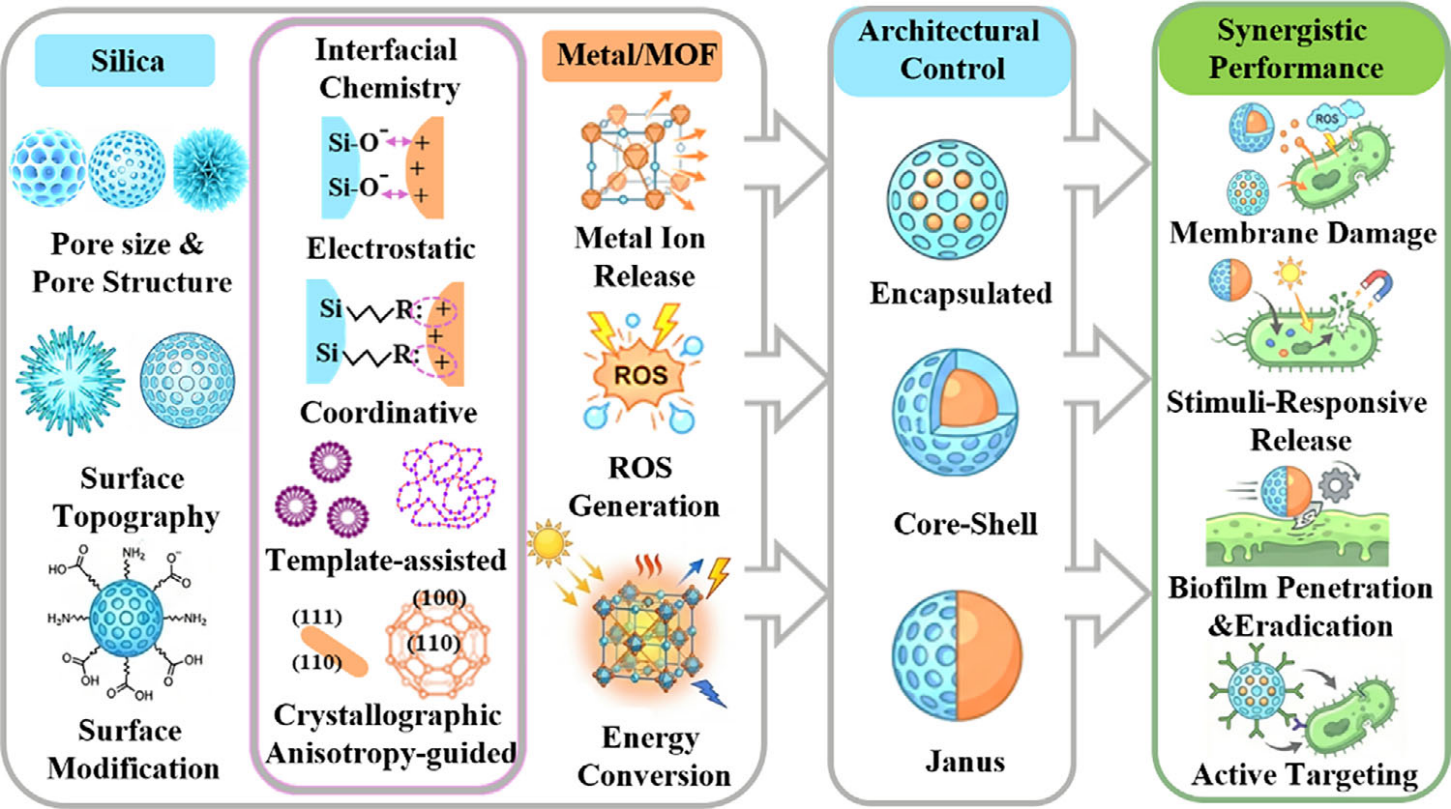
Schematic illustration of interfacial chemical toolbox for constructing silica&metal‐based heterostructures and their antibacterial application.

This review establishes interfacial chemistry as the central design principle to achieve well‐defined silica&metal‐based nanoarchitectures with synergistic antibacterial properties. We first elucidate how tailored interfacial chemistry, including electrostatic, coordinative, template‑assisted, and crystallographic anisotropy‑directed assembly strategies overcome inherent physicochemical incompatibilities (e.g., significant lattice mismatch and inherently weak interfacial affinity) to enable the construction of versatile heterostructures. In particular, we emphasize that the silica component acts not merely as a passive carrier but as an active structural and functional modulator. Its integration mitigates the physiochemical limitations of metal‐based materials and enables desired functionality. Subsequently, we examine how these engineered nanohybrids achieve synergistic functional performance in four major antimicrobial applications, including direct membrane disruption, stimuli‑responsive activation, biofilm penetration/eradication and receptor‐mediated active targeting to pathogenic bacteria (Figure 1). Finally, our perspectives on the challenges and prospects of interfacial engineering, material synthesis and clinical translation requirements for silica&metal‐based materials will be discussed.

## 2 Interfacial Chemistry‐Directed Heterostructure Construction

This section moves beyond synthetic strategies to the fundamental chemical principles governing the formation of silica&metal‐based nanohybrids. We specifically focus on the synthetic chemistry that dictates interfacial bonding and ultimate nanoarchitecture (Table 1).

**TABLE 1:** A toolbox of interfacial chemistry for engineering silica&metal‐based nanohybrids, their corresponded nanoarchitecture and functional outcome/benefits contributed by silica.

Interfacial Strategy ^a^	Types of Nanohybrid ^b^	Nano‐Architecture	Outcome/Benefits Contributed by Silica	Refs.
1.Native silanol‐mediated assembly				
Electrostatic interaction	Ag NPs@mSiO_2_; (Pt/ Ru/ Rh) NPs@mSiO_2_; Pd‐CoO NPs@mSiO_2_	Encapsulated within pores	Confines and disperses NPs within mesopores, maximizes accessible surface area, enhances catalytic/antibacterial performance.	[38, 39, 40]
Coordinative interaction	Fe_2_O_3_@SBA‐15 and Fe^3+^ ‐loaded DMSNs	Encapsulated within pores	Stabilizes Fe species and prevents their aggregation	[39, 42]
	MOF‐5@mSiO_2_		Directs heterogeneous nucleation and oriented growth of MOF crystals.	[44]
	ZIF‐8@mSiO_2_; Cu‐BTC MOF@mSiO_2_		Confines MOF growth within large pores to achieve high metal loading and prevent crystal aggregation.	[45, 46]
	Cu_2_O NPs@mSiO_2_@TA‐Cu	Hierarchical core‐shell	Provides a porous scaffold for confined growth and a multi‐layered interface enabling synergistic, acid‐triggered Fenton‐like activity.	[43]

2. Functionalized surface‐mediated assembly				
Thiol (‐SH) coordinative interaction	Au NPs@mSiO_2_	Encapsulated within pores	Strong surface coordinative bonding within pores yields ultrafine, stable NPs with high accessible active sites for efficient energy‐conversion.	[49]
Amine (‐NH_2_) coordinative interaction	ZnO NPs@mSiO_2_		Selectively coordinates precursors to confine ZnO growth exclusively within mesopores.	[51]
	SBA‐15@HKUST‐1	Core‐shell	Serves as structure‐directing agent to induce oriented growth of MOF with reduced particle sizes and hierarchical porous architectures.	[52]
Carboxyl (‐COOH) coordinative interaction	ZIF‐8/ZIF‐67/MIL‐88/MOF‐74@mSiO_2_		Improves stability of MOF and overall dispersion of nanohybrid.	[53]
Amine/ methyl (‐NH_2_ / ‐CH_3_) electrostatic interaction	CuO NPs@SBA‐15	Encapsulated within pores	Differential surface functionalization spatially confines CuO growth within internal pores, enhancing loading amount and preventing external aggregation.	[50]
Amine /sulfonic acid (‐NH_2_ / ‐SO_3_H) electrostatic interaction	Ag/Pt NPs@mSiO_2_@ZIF‐8/ZIF‐67	Hierarchical core‐shell	SiO_2_ shell stabilizes metal cores, enhances dispersion, and provides a charged interface for growth of MOF shell.	[54]

3. Soft template‐assisted interfacial assembly				
CTAB‐assisted electrostatic interaction	Au nanorod@mSiO_2_	Core‐shell	Improves core stability; adjustable shell thickness regulates fluorescence efficiency.	[55]
	Co_3_O_4_ NPs@mSiO_2_		Maintain small core sizes; adjustable shell thickness regulates mass diffusion rate.	[57]
	MIL‐101Cr@mSiO_2_		Facilitates the diffusion of hydrophobic reactants and improves the stability of the core	[62]
	Au nanorod@mSiO_2_@Fe‐based MOF	Core‐shell‐shell	Serves as the intermediate shell for growth of MOF and loading of therapeutic drugs.	[56]
	ZIF‐67 or Co‐CN@mSiO_2_	Core‐shell or York‐shell	Facilitated mass transfer through mesoporous pore channel and stabilizes the inner core.	[60]
	ZIF‐8@mSiO_2_		Protects inner core and provides a matrix to encapsulate bio‐cargoes.	[61]
CTAB mediated in situ dynamic assembly	Fe_3_O_4_ NPs@ mSiO_2_	Core‐shell	Prevents the aggregation of inner core and serves as drug carrier for controlled release.	[58]
CTAB mediated kinetic‐controlled assembly	BaTiO_3_ NPs@mSiO_2_	Patchy Janus	Tunable silica coverage provides programmable protection and interfacial functionality.	[59]
PVP assisted coordinative interaction	Au/Ag NPs@SiO_2_; Mn‐based MOF@SiO_2_	Core‐shell	Stabilizes the core and facilitates surface functionalization with therapeutic molecules.	[66, 70]
PVP/CTAB assisted coordinative/electrostatic interaction	CuO/MgO/M_x_O_y_ NPs@mSiO_2_		Improves stability and dispersity of inner core; the mesoporous structures promote mass transfer and catalytic reaction.	[67, 68, 69]

4. Hard template‐assisted spatial confined assembly				
PS sphere as physical masking	α‐Fe_2_O_3_ NPs@SiO_2_	Dumbbell‐like Janus	Establishes a well‐defined substrate for site‐selective nucleation and growth of secondary functional materials.	[71]
RF polymer as nucleophilic interface	Fe_3_O_4_ NPs@mSiO_2_	Cauliflower‐like core‐shell	Surface topology enhances interfacial nano‐bio interactions.	[72]
	Fe_3_O_4_@ SiO_2_@RF&PMOs	Multiple pod‐like core‐shell		[73]
SiO_2_ as protective & nucleation interface	Fe_3_O_4_ NPs@mSiO_2_	Core‐shell	Intermediate dense SiO_2_ layer protects inner core from degradation and serves as nucleation interface for outer growth of mesoporous silica.	[74]

5. Crystallographic anisotropy‐guided assembly				
CTAB mediated site‐selective assembly	Au nanorod@mSiO_2_	Dumbbell‐like Janus	Enables anisotropic, site‐selective silica coating for constructing complex plasmonic nanostructures.	[75]
Facet‐selective coordinative assembly	ZIF‐8&mSiO_2_	Accropode‐like Janus	Janus mesoporous SiO_2_ shell enhances the bio‐adhesion and therapeutic cargo loading.	[78]
Facet‐selective coordination & etching	ZIF‐8&mSiO_2_ or ZIF‐8&mSiO_2_&MOFs	Anisotropic Janus	Acts as interface mediators for precise morphological evolution such as hexapods, nested nanocages, and octopods.	[79]

^a^ CTAB: Cetyltrimethylammonium bromide;

^b^ mSiO_2_: Mesoporous silica nanoparticle; SBA‐15: Santa Barbara Amorphous‐15; DMSNs: Dendritic mesopore silica nanoparticles**;** Cu‐BTC: Copper benzene‐1,3,5‐tricarboxylate; HKUST‐1: Hong Kong University of Science and Technology‐1; RF: resorcinol‐formaldehyde resin; PMO: periodic mesoporous organosilica.

### 2.1 Innate Surface Chemistry: Silanol Group‑Mediated Assembly

The most fundamental strategy in interfacial engineering exploits the intrinsic chemistry of silica, governed by its abundant surface silanol (Si‐OH) groups. These groups exhibit bimodal acid‐base behavior, characterized by two distinct acid dissociation constants (pKa ≈ 4.5 for 15–20% of sites and pKa ≈ 8.5 for 80–85% of sites) [37], which enables two major interfacial interactions for anchoring metal precursors: (1) electrostatic interaction via pH‑dependent deprotonation to generate silanolates (Si‐O^−^), and (2) weak coordinative interaction via the Lewis‑basic oxygen lone pairs. Subsequent processing, such as reduction, oxidation, or thermal treatment transforms the anchored species into spatially confined metal nanoparticles or promotes heterogeneous nucleation and crystallization of MOFs via adsorption of organic linkers.

#### 2.1.1 Electrostatic Interaction Assisted Anchoring and Confinement

**FIGURE 2:**
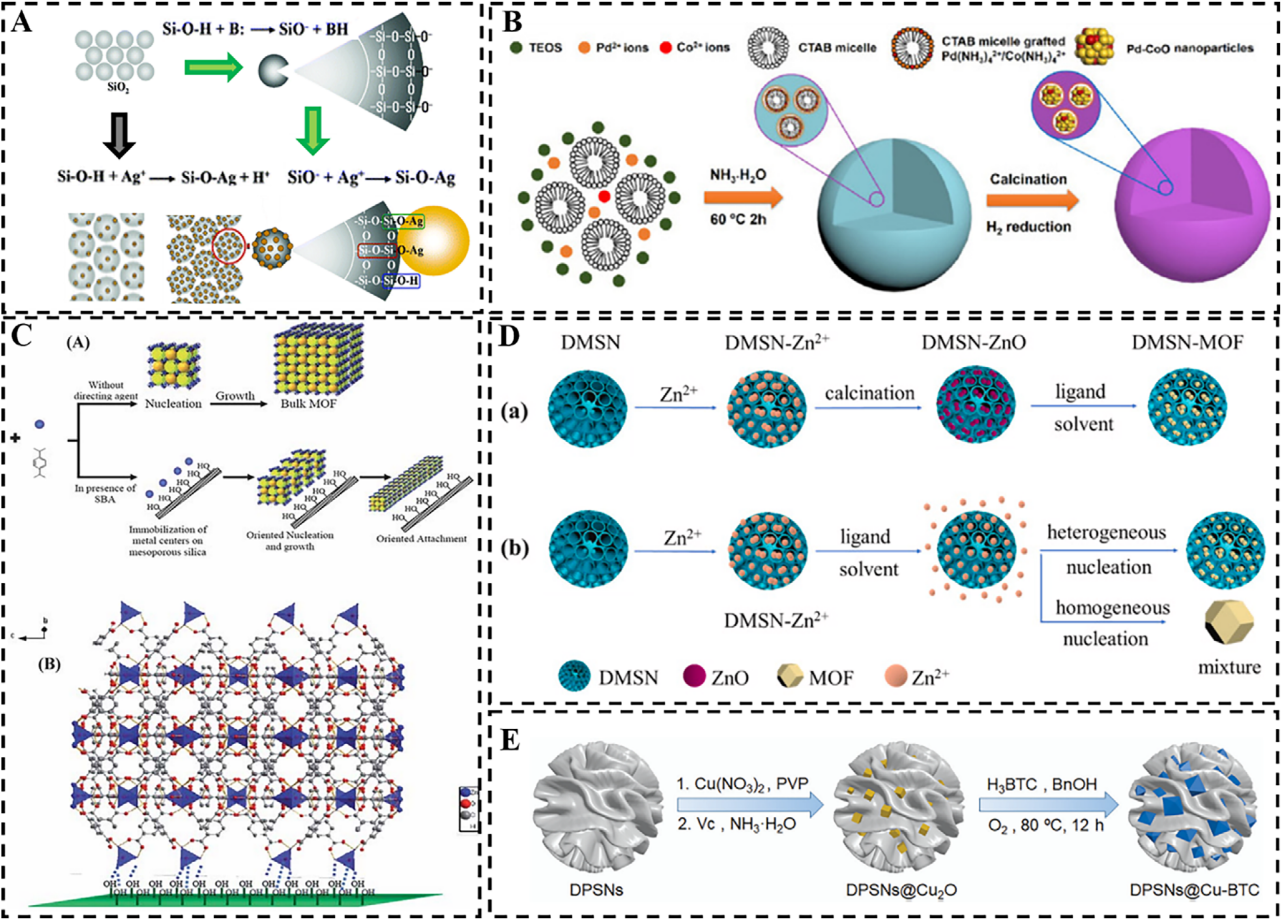
Silanol group mediated interfacial chemistry: (A) Ag NPs anchored on the surface of silica nanoparticle, with (Green Route) and without (Black Route) deprotonation of silanol group. Reproduced with permission [38] Copyright 2019, ACS Publications. (B) Bimetallic Pd‐CoO NPs encapsulated within the MSNs via metallomicelle templated self‐assembly. Reproduced with permission [41] Copyright 2019, Wiley. (C) Formation mechanism of MOF‐5 on the surface of SBA‐15 and corresponded interfacial chemical reaction. Reproduced with permission [44] Copyright 2013, The Royal Society of Chemistry. (D) ZIF‐8 confined growth within DMSN with high content via a metal oxide conversion strategy. Reproduced with permission [45]. Copyright 2023, Elsevier. (E) Cu_2_O induced in situ growth of Cu‐BTC within DPSNs. Reproduced with permission [46]. Copyright 2020, The Royal Society of Chemistry.

Electrostatic attraction provides a straightforward anchoring pathway. Increasing the solution pH above the pKa values of silanol groups induces deprotonation, generating negatively charged silanolates (‐O^−^) that strongly interact with positively charged metal precursors. For instance, Kang et al. [38] deprotonated silanol groups into nucleophilic silanolates using alkaline catalysts, which significantly intensified electrostatic interaction with Ag^+^ ions (Figure 2A). After in situ formation of nanoparticles within the mesopore, the content of silver increased from 2.00% to 4.31%. Similarly, Kleitz et al. [39] explored this pH‐dependent electrostatic interaction strategy to anchor Fe^3+^ species into DMSNs. By elevating the pH value to basic conditions, the deprotonated surface silanol groups with negative charge attract Fe^3+^ ion, yielding Fe^3+^ deposited DMSNs. The immobilized Fe^3+^ sites could further coordinate with tannic acid. This coordination interaction significantly enhanced the colloidal stability of the Fe^3+^ containing DMSNs, beneficial for their applications in biomedical fields.

In addition to serving as static anchoring sites, silanol groups can be generated in situ during silica condensation, enabling one‐pot encapsulation of metal species into the growing silica matrix. For instance, Zeng et al. [40] reported a one‐pot co‐assembly route to integrate ultrasmall (∼1 nm) noble metal nanoparticles (Pt, Ru, Rh) into the pore cavities of MSNs. This approach employed cationic surfactants, which initially assembled with pre‐formed metal nanoparticles to form positively charged metal‐micelle composites. These composites then electrostatically interacted with negatively charged hydrolyzed silica precursors, inducing silica condensation around the micelles and ultimately confining the nanoparticles within the mesoporous silica framework. The obtained material exhibited highly accessible catalytic sites, leading to superior catalytic performance.

Yan et al. [41] further extended this strategy to encapsulate bimetallic Pd‐CoO nanoparticles within MSNs using CTAB‐templated micelles. During this process, ammonia first chelated with Pd and Co precursors to form Pd (NH_3_)_4_^2+^ and Co (NH_3_)_6_^3+^ complexes, which subsequently co‐assembled with CTAB. The above positively charged metallomicelles then electrostatically interacted with negatively charged hydrolyzed silica precursors, leading to the incorporation of the metal complexes into the growing silica matrix (Figure 2B). After the process of calcination and reduction, ultrafine Pd‐CoO nanocrystals were confined within the mesopores of MSNs, effectively preventing nanoparticles from aggregation and sintering under harsh conditions.

#### 2.1.2 Coordinative Interaction Assisted Nucleation and Growth

Beyond electrostatic forces, surface silanol groups also function as Lewis‑base sites capable of forming coordinative bonds with Lewis‑acidic metal ions (e.g., Fe^3+^, Zn^2+^, Cu^2+^). For instance, Shi et al. [42] introduced highly dispersed iron species into SBA‐15 via a co‐impregnation method, reslting in Fe_2_O_3_ nanoclusters within the mesoporous channels and isolated Fe^3+^ ions incorporated into the silicate framework. The abundance of silanol groups on the pore surface enabled a coordinative interaction with iron precursor, allowing for in situ site anchoring and preventing aggregation during thermal treatment. Increasing the loading amount of Fe facilitated the formation of Fe_2_O_3_ nanocluster. This strategy was also demonstrated to fabricate mangansese Mn_x_O_y_@SBA‐15.

Moving forward to hierarchical interfacial design, He et al. [43] developed a Cu^2+^‐loaded mesoporous silica nanoparticle in which silanol groups coordinated with copper precursor, enabling pore‐confined deposition of CuO_2_ via in situ oxidation. Tannic acid was subsequently deposited through hydrogen bonding with surface silanol groups and further coordinated with Cu^2+^ to form an outer tannic acid‐copper network. This multi‐layered interfacial architecture achieved acid‐triggered Cu^2+^ release, amplifying chemodynamic therapy via synergistic Fenton‐like catalysis.

This silanol group mediated coordinative interaction also serves as a foundation for integrating MOFs, which was demonstrated by Morsali et al. [44]. In their work, silanol groups within the SBA‐15 served as directing agents for the crystallization and oriented growth of MOF‐5. This process was initiated by Zn^2+^‐silanol coordinative interaction, followed by linkages between metal centers and organic ligands. Additionally, hydrogen bonding between surface silanol groups and oxygen‑rich coordination environment of zinc clusters enhanced interaction between the mesoporous silica surface and the growing of MOF microcrystals (Figure 2D). In contrast, passivation of silanol groups with ‐Si(CH_3_)_3_ groups completely inhibited the formation of MOF‐5, confirming a direct coordination‐driven mechanism from silanol groups.

Building upon this principle, Yu et al. [45] pioneered a metal oxide conversion strategy to achieve high loading of MOFs within silica matrices. Zn^2+^ ions were first encapsulated within DMSNs via coordination interaction and calcined to form solid ZnO nanoparticles. These nanoparticles then gradually released Zn^2+^ to react with 2‐methylimidazole, leading to the heterogeneous nucleation and growth of ZIF‐8 within the pores. The resulting DMSN@ZIF‐8 composites achieved exceptionally high Zn/Si molar ratios (0.4–0.8:1), far exceeding those from conventional impregnation (Figure 2E). Similarly, Du et al. [46] confined Cu_2_O nanoparticles within DMSNs via coordination interaction between Cu^2+^ and silanol groups with in situ reduction. Subsequently, these Cu_2_O nanoparticles slowly dissolved in an acidic oxidative medium, releasing Cu^2+^ ions for the direct formation of Cu‐BTC with benzene‐1, 3, 5‐tricarboxylic acid (H_3_BTC) ligands (Figure 2F).

### 2.2 Engineering the Interface: Functionalized Surface‐Directed Assembly

While the innate chemistry of surface silanol groups provides a simple route for interfacial assembly, their inherently weak and non‐selective interactions often result in limited metal loading capacities and spatial distribution. To overcome these limitations, the silica surface can be deliberately functionalized through covalent grafting of organic functional groups (e.g., ‐NH_2_, ‐SH, ‐COOH). This modification transforms silica surface into a chemically programmable interface, where interfacial affinity and specificity are dramatically enhanced according to hard‑soft acid‑base principles [47, 48]. This section explores how such tailored surfaces dictate the precise localization, enhanced loading efficiency, and controlled morphology of integrated metallic‐based components.

#### 2.2.1 Engineered Surface for Loading of Metal/Metal Oxide

Pre‐functionalization with organic ligands provides stronger and more selective binding sites for metal ions, enabling controlled loading, dispersion, and spatial location of metallic nanocomponents. For instance, thiol (‐SH) groups, which act as soft Lewis bases, can form strong covalent bonds with soft Lewis acids like Au^3+^. Shi et al. [49] functionalized hollow mesoporous silica with ─SH to covalently bond with Au^3+^ ions, which were subsequently reduced in situ to form ultrafine, uniformly distributed Au nanoparticles. This precise control over size and distribution yielded a high surface area to volume ratio, which was beneficial for enhanced photothermal therapy.

**FIGURE 3:**
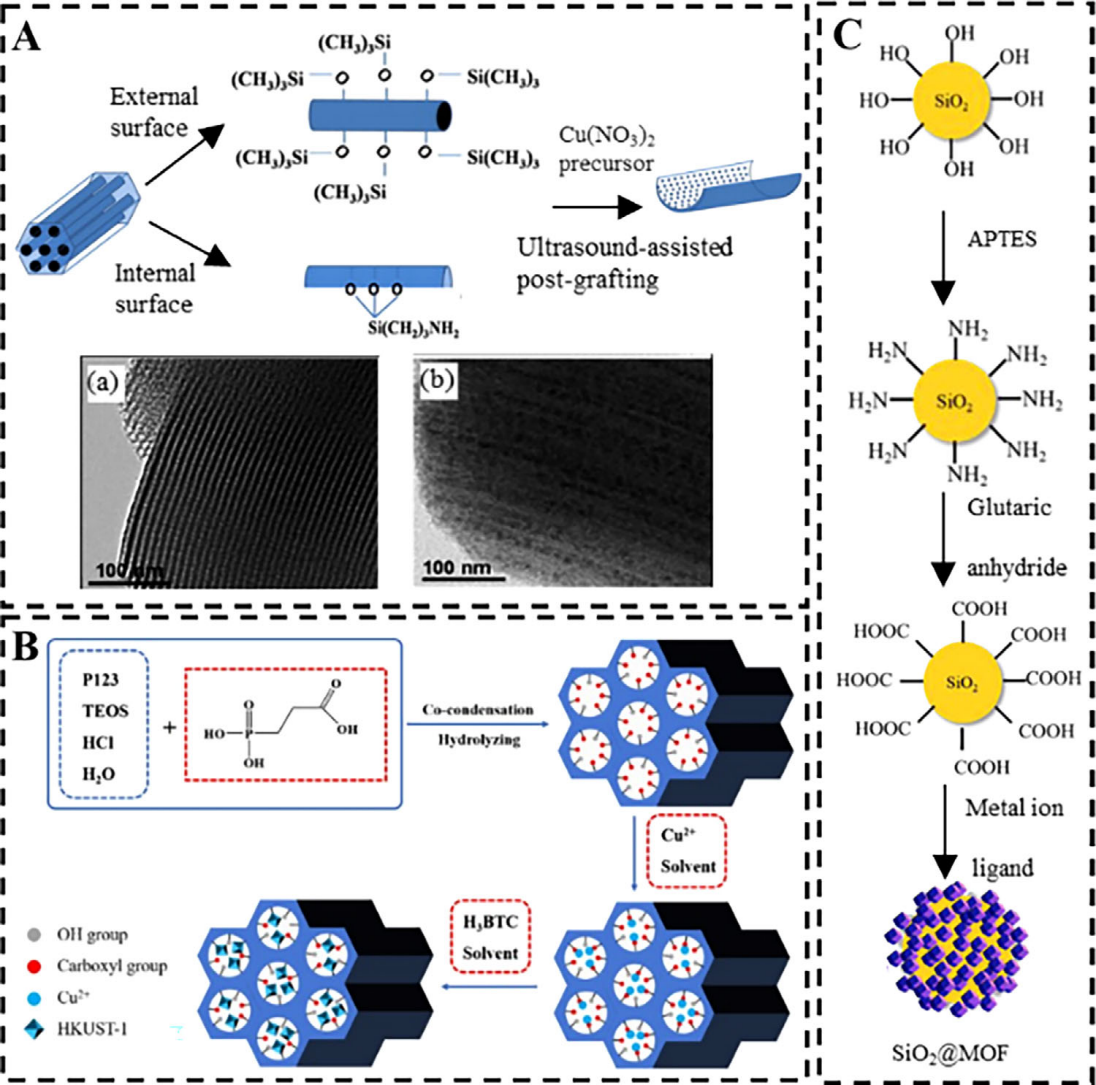
Functionalized silica mediated interfacial chemistry: (A) Synthesis procedure of CuO/SBA‐15 and HRTEM images of (a) SBA‐15, (b) CuO/SBA‐15. Reproduced with permission [50]. Copyright 2013, Elsevier. (B) Synthesis process of HKUST‐1@SBA‐15 nanohybrid. Reproduced with permission [52]. Copyright 2022, Elsevier. (C) Carboxylic acid functionalized silica induced the epitaxial growth of MOFs. Reproduced with permission [53]. Copyright 2022, ACS Publications.

This strategy is equally effective for synthesizing spatial confined metal oxide nanoparticles via a “anchoring‐calcination” pathway. For example, amine (─NH_2_) groups belong to hard Lewis base exhibit strong affinity for borderline Lewis acid Cu^2+^ ions. Shi et al. [50] modified the outer and inner surfaces of SBA‐15 with ─CH_3_ and ─NH_2_ groups, respectively. The outer ‐CH_3_ groups effectively restrained the growth of CuO outside the pore channel after calcination. In contrast, ─NH_2_ groups with coordinative affinity with Cu^2+^ ion increased both the loading amount and dispersion of CuO on the internal porous surface (Figure 3A). Similarly, Yan et al. [51] functionalized MCM‐41 with ethylenediamine, whose nitrogen lone pairs as a hard Lewis base, selectively coordinated with borderline Lewis acid Zn^2+^ ions. Subsequent calcination in air transformed these coordinated complexes into ZnO nanoparticles exclusively confined within the mesopores. This spatial distribution was directed by the preferential binding of precursors to the high‐internal‐area pore walls.

#### 2.2.2 Directed Growth of MOF on Engineered Surface

Pre‑functionalization of silica surfaces also provides a powerful route to direct the heterogeneous nucleation and oriented growth of MOFs, offering superior control over crystallinity, interfacial stability, and final architecture compared to native silanol groups. For example, Shi et al. [52] functionalized the inner pore surfaces of SBA‐15 with carboxyl groups to enhance the coordination with Cu^2+^ ions. Subsequent immersion in a carboxylate ligand solution led to the confined growth of HKUST‐1 within the mesopores (Figure 3B). The synergistic effects SBA‐15 and in situ formed HKUST‐1 endowed the composite material with hierarchical pore structure and ensured uniform distributions of Cu‐based MOFs within the silica support. In another example, Liu et al. [53] employed carboxyl‐functionalized Stöber silica to coordinate with various metal ions (Zn^2+^, Co^2+^, Fe^3+^) on the outer surface, followed by the adsorption of organic ligands, forming a protective MOF shell (ZIF‐8, ZIF‐67, MIL‐88). This core‐shell structure improved the core stability and overall dispersion of the resulted nanohybrid (Figure 3C).

This functionalization strategy can be extended to construct more complex multi‐component hybrids. In the construction of multi‑component metal@silica@MOF nanohybrids, Zeng et al. [54] first confined metal nanoparticles (e.g., Pt, Ag) within the amine‐functionalized mesopore channels of silica matrix via electrostatic interaction to prevent their aggregation. The external silica surface was then sequential modified with amine and sulfonic acid groups to generate a negatively charged surface. This engineered interface electrostatically attracted Zn^2+^ ions, initiating the oriented growth of a ZIF‐8 or ZIF‐67. In this architecture, the functionalized silica interface played a dual mediating role: it stabilized the encapsulated metal nanoparticles and induced the oriented growth of MOF as the outer shell, achieving synergistic multifunctional nanohybrids.

### 2.3 Programmable Assembly via Interfacial Templates

The precise morphological control such as uniform core‑shell, anisotropic Janus, or yolk‑shell structures can be achieved by introducing exogenous molecular or supramolecular templates at the interface to guide programmable assembly, which can be classified into two paradigms: (1) soft templating, using surfactants for electrostatic guidance or ligands for coordinative assembly; and (2) hard templating, typically involving sacrificial colloidal particles or preformed layers to impose physical confinement or protection. These strategies enable precise regulation of critical physicochemical parameters such as shell thickness, porosity, and anisotropy.

#### 2.3.1 Soft Templating via Surfactant‐Mediated Assembly

**FIGURE 4:**
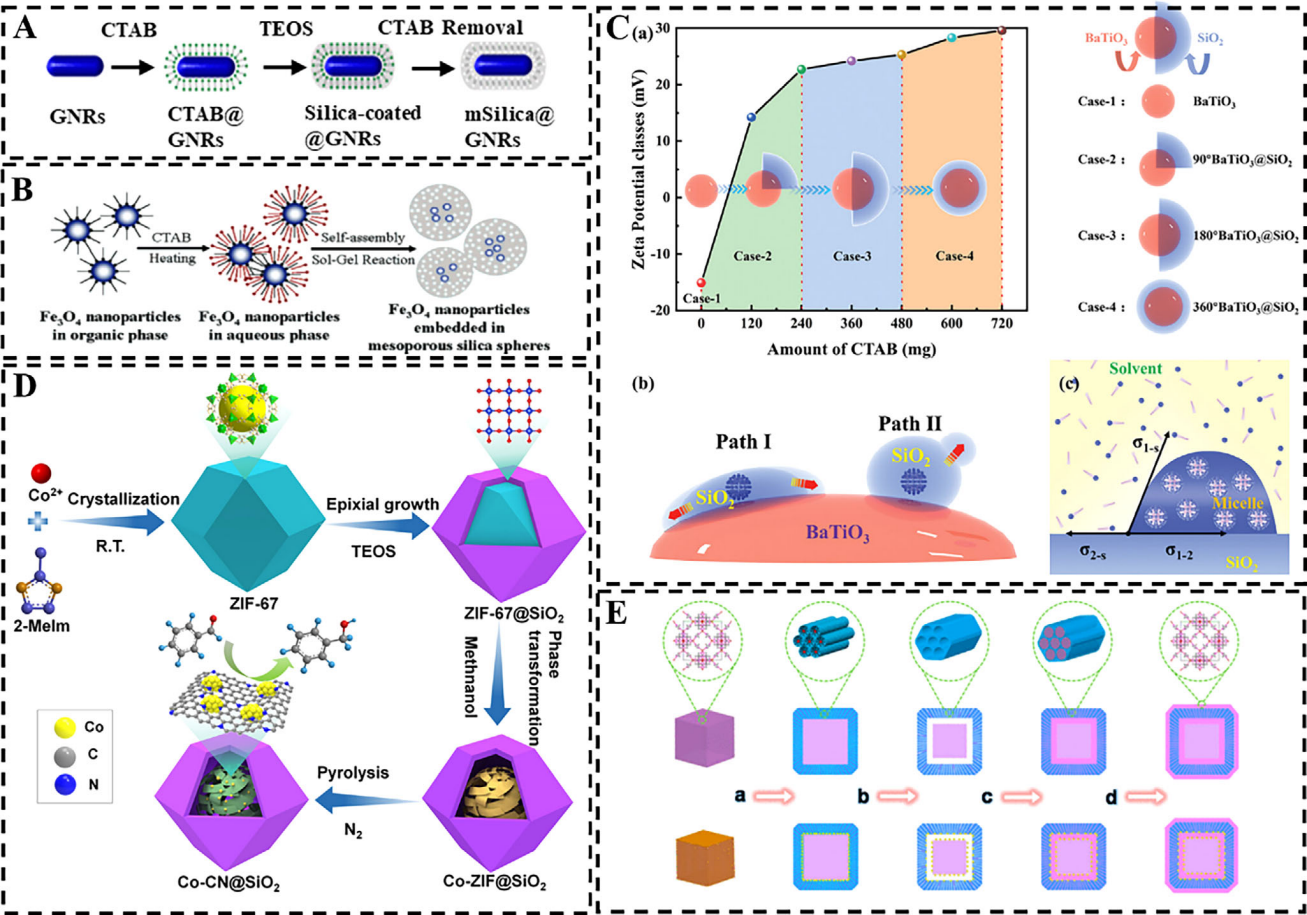
Surfactant‐templated interfacial chemistry via electrostatic interaction: (A) Core‐shell Au nanorod@mSiO_2_. Reproduced with permission [56]. Copyright 2023, ACS Publications. (B) Monodisperse magnetite nanocrystals embedded in MSNs. Reproduced with permission [58]. Copyright 2005, Wiley. (C) Asymmetric BaTiO_3_@SiO_2_ NPs via surface kinetic‐controlled growth of silica. Reproduced with permission [59]. Copyright 2024, Wiley. (D) Structural evolution from core‐shell ZIF‐67@mSiO_2_ into yolk‐shell Co‐CN@SiO_2_. Reproduced with permission [60]. Copyright 2025, ACS Publications. (E) Structural transformation between core‐shell and yolk‐shell type of ZIF‐8@mSiO_2_. Reproduced with permission [61]. Copyright 2014, ACS Publications.

This strategy employs cationic surfactants such as cetyltrimethylammonium bromide (CTAB) to direct the electrostatic assembly of hydrolyzed anionic silica precursors onto target cores. CTAB serves a dual role: stabilizing the colloids by replacing surface anionic stabilizers while providing a uniform cationic interface that templates continuous silica shell deposition. This is particularly critical for coating noble metal nanoparticles (e.g., Au, Ag), which are typically stabilized by anionic capping agents (e.g., citrate) that electrostatically repel silica precursors. For example, Kim et al. [55] employed CTAB‐capped gold nanorods as the core to construct a plasmonic mesoporous silica platform (Figure 4A). The silica shell was subsequently functionalized with carboxyl groups for antibody conjugation and loaded with aminated Cy5 fluorophores. By tuning the shell thickness, plasmon‐enhanced fluorescence could be modulated to achieve ultrasensitive virus detection. Similarly, Zhang et al. [56] adopted a comparable approach to obtain gold nanorods@mSiO_2,_ which served as the core for constructing a multi‐component nanohybrid through muti‐interfacial assembly. After silica shell functionalized with carboxyl groups, which acted as the Lewis hard acid, they coordinated with Fe^3+^ ions as Lewis borderline base, forming a Fe‐based MOF as the outer shell in the presence of organic ligands. Therapeutic drugs were further loaded into the mesoporous structures of silica shell, allowing for controlled release and integrating multifunctionality into one nanohybrid.

This strategy extends to metal oxide cores with inherent negative surface charges. For instance, the adsorption of CTAB onto negatively charged Co_3_O_4_ cores formed a cationic surface to electrostatically condense with anionic silica precursor, initiating shell growth [57]. This process could be self‐sustaining, where progressive silica deposition increased surface negativity, thereby driving further CTAB adsorption and enabling controlled shell thickening. Similarly, Hyeon et al. [58] employed the amphiphilic CTAB to transfer the hydrophobic magnetic nanoparticles (Fe_3_O_4_) into aqueous solution. In this process, the hydrophobic tails of CTAB anchored to the Fe_3_O_4_ surface while the exposed cationic head electrostatically directed the condensation of hydrolyzed silica precursors, ultimately yielding monodisperse core‐shell structures for subsequent drug loading (Figure 4B). Precise manipulation of interfacial interactions further allows for kinetic control over shell morphology. Zhang et al. [59] tuned the concentration of CTAB to control the interfacial nucleation and growth kinetics of co‐assembled silica on the surface of BaTiO_3_ core. A low CTAB concentration led to sparse, patchy coverage of silica islands (90°), while a higher concentration promoted more extensive heterogeneous nucleation and subsequent lateral growth, resulting in either semi‐encapsulation (180°) or a complete silica shell (360°) (Figure 4C).

The electrostatic templating principle is also applicable to crystalline MOF cores. Liu et al. [60] employed CTAB as a cationic surface mediator to direct the electrostatic assembly of hydrolyzed silica precursors onto ZIF‑67 nanoparticles, forming a uniform mesoporous silica shell. Subsequent solvent‐thermal treatment and calcination triggered the in situ transformation of the ZIF‑67 core into Co nanocrystals supported on nitrogen‑doped carbon nanosheets (Co‐CN), thereby converting the core‐shell into a yolk‐shell structure. The mesoporous silica shell not only promoted mass transport to enhance reaction kinetics but also served as a protective shell to stabilize the inner core (Figure 4D). Similarly, Zeng et al. [61] synthesized core‐shell ZIF‐8@mSiO_2_ via CTBA assisted interfacial interactions. The subsequent calcination removed the surfactant template and induced partial infusion and contraction of ZIF‐8 core, yielding a yolk‐shell structure. Interestingly, this structure could be reconverted back into a core‐shell morphology via precursor infiltration and re‐growth within the mesoporous shell, showing dynamic structural control (Figure 4E). Extending this approach to surface property modulation, Janiak et al. [62] synthesized core‐shell structured MIL‐101Cr@mSiO_2_ nanoparticles in which the ultrathin silica shell replicated the rough surface of the inner irregular MOF nanoparticle. This rough surface turned the typically hydrophilic silica shell into a hydrophobic coating, which facilitated the diffusion of hydrophobic reactants and enhanced the catalytic activity of the MOF core.

#### 2.3.2 Soft Templating via Ligand‐Mediated Assembly

**FIGURE 5:**
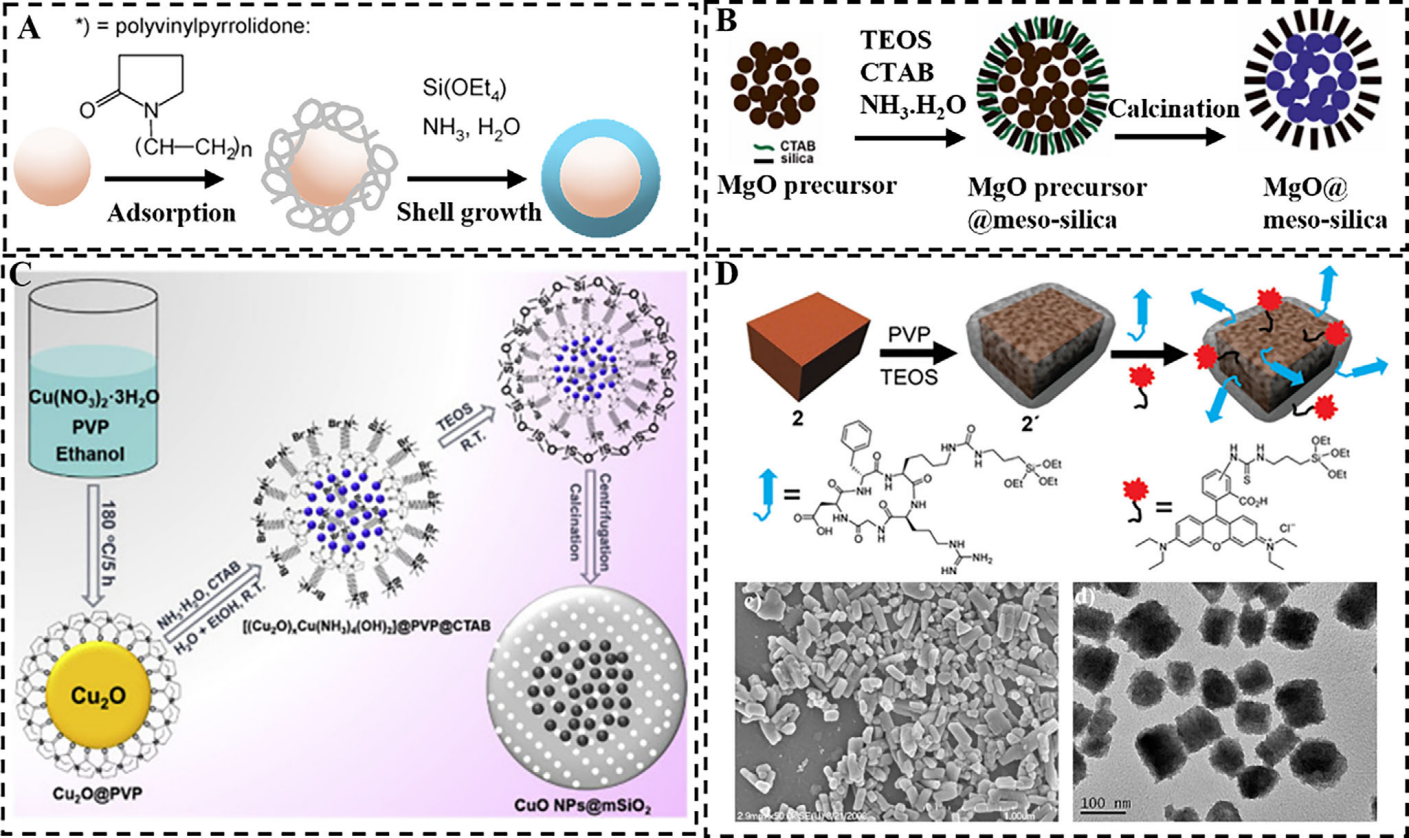
Ligand‐templated interfacial engineering via coordinative chemistry: (A) A general method for synthesizing core‐shell metal/metal oxide NPs@SiO_2_ using PVP as a surface primer. Reproduced with permission [66]. Copyright 2003, ACS Publications. (B) Core‐shell structured MgO@mSiO_2_ fabricated via PVP stabilized MgO core and CTAB templated silica shell. Reproduced with permission [68]. Copyright 2015, Royal Society of Chemistry. (C) Watermelon‐like core‐shell type of CuO@mSiO_2_ NPs fabricated via PVP stabilized Cu_2_O core and CTAB templated silica shell. Reproduced with permission [69]. Copyright 2020, Elsevier. (D) Core‐shell type of Mn‐based MOF@SiO_2_ functionalized with a fluorophore and a cell‐targeting peptide along with corresponding SEM images of the pristine Mn‐based MOF and the Mn‐based MOF@mSiO_2_ composite. Reproduced with permission [70]. Copyright 2008, ACS Publications.

This approach utilizes molecular ligands such as polyvinylpyrrolidone (PVP), which acts as an interfacial primer. The carbonyl groups of PVP coordinate with unsaturated metal sites on metal or metal oxide cores (e.g., Au, Ag, CuO, MgO) via Lewis acid‐based interaction [63]. The obtained PVP‐mediated interface not only stabilizes the colloid against aggregation but actively promotes localized silica deposition via hydrogen‐bonding interactions between silanol groups and the pyrrolidone carbonyl groups (C═O) [64, 65]. Van Blaaderen et al. [66] pioneered this approach, developing a general method to coat colloidal nanoparticles such as gold and silver with a uniform silica shell by employing PVP as a mediating layer (Figure 5A).

Ligands can also function synergistically with other structure‑directing agents. A PVP layer stabilizing a metal oxide core can co‐assemble with CTAB micelles via hydrophobic interaction. The hydrophilic head of CTBA then electrostatically directs the condensation of hydrolyzed anionic silica precursors to form a mesoporous silica shell. This cooperative mechanism was exemplified by CuO@SiO_2_ and MgO@SiO_2_ core‐shell structures reported by Song et al. [67] and Yu et al. [68], respectively (Figure 5B). Notably, the CuO and MgO cores in these systems consist of nanocrystals with small particle sizes (∼8 nm and ∼4 nm, respectively), whose nanoscale dimensions enhanced catalytic activity through high specific surface area and quantum confinement effects. An advanced extension of this synergistic co‐assembly was demonstrated by Fan et al. [69], who developed a general encapsulation strategy for various metal oxides (e.g., Fe_2_O_3_, Co_3_O_4_, ZnO) within a mesoporous silica shell. Taking watermelon‐like CuO@mSiO_2_ as an example, ammonia treatment transformed the PVP‐stabilized Cu_2_O nanoparticles into ultrasmall coordination complexes ([(Cu_2_O)_x_Cu(NH_3_)_4_(OH)_2_]) while maintaining the PVP coating. CTAB micelles then co‐assembled with this PVP modified interface and directed silica condensation around the core via electrostatic interaction. The subsequent calcination generated discrete CuO nanoparticles (∼4 nm) confined within the mesoporous silica shell, maximizing the accessibility of catalytic active sites (Figure 5C).

The ligand‑mediated assembly principle extends to MOFs. PVP pre‐functionalized MOF creates a protective layer that effectively protecting the MOF framework from hydrolysis under the alkaline conditions required for silica deposition. For instance, Lin et al. [70] modified the surface of Mn‐based nanoscale MOFs (NMOFs) with PVP. During the subsequent sol‐gel process, hydrogen bonding between the silanol groups and the carbonyl groups of the PVP chains facilitated the deposition of a thin silica layer onto the MOF core (Figure 5D). The obtained silica shell effectively stabilized the NMOFs core against dissolution, enabled site‐specific delivery and release of Mn^2+^ ions. This provided a versatile platform for further functionalization, making it suitable for targeted magnetic resonance imaging and therapy.

#### 2.3.3 Hard Templating via Spatial Confined Assembly

In addition to soft molecular templates, spatial confinement can be achieved by hard, sacrificial templates or through precise chemical engineering of the core's interfacial properties. This approach allows for the fabrication of asymmetric silica shells by selectively masking parts of the core or by controlling nucleation sites through tailored surface chemistry.

**FIGURE 6:**
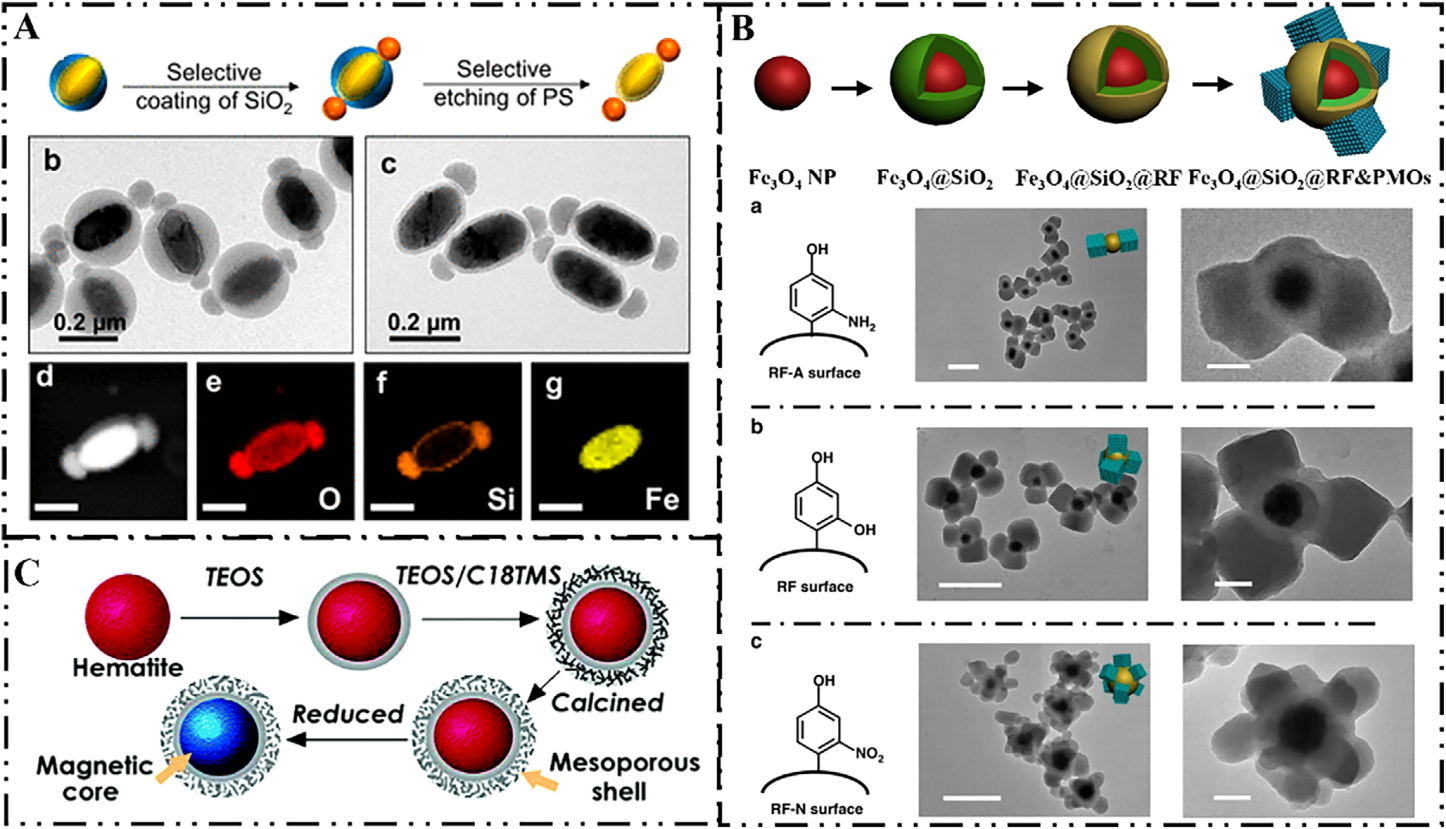
Interfacial engineering via hard templating assisted spatial confinement: (A) Dumbbell‐like α‐Fe_2_O_3_@SiO_2_ nanoparticles obtained via physical masking by PS template. Reproduced with permission [71]. Copyright 2019, ACS Publications. (B) Fe_3_O_4_@SiO_2_@RF&PMOs with multipods surface topographies obtained via surface‐kinetic mediated multi‐site nucleation. Reproduced with permission [73]. Copyright 2019, Springer Nature. (C) Core‐shell type of Fe_3_O_4_@mSiO_2_ nanosphere obtained via core‐protective hard template. Reproduced with permission [74]. Copyright 2005, ACS Publications.

Physical masking utilizes sacrificial barriers to protect specific regions of the core, restricting silica growth to exposed surfaces. He et al. [71] synthesized dumbbell‐like α‐Fe_2_O_3_@SiO_2_ heterostructures using polystyrene (PS) as a sacrificial template. After depositing an initial silica layer on PVP‐modified α‐Fe_2_O_3_ via coordinative interaction, PS preferentially grew on the lower curvature regions. The subsequent condensation and polymerization of silica were confined to the exposed higher curvature tips, leading to the final formation of anisotropic morphology (Figure 6A). This versatile hard‐templating strategy was successfully extended to nonspherical colloidal seeds, including rod‐shaped, peanut‐shaped α‐Fe_2_O_3_ and FeOOH nanorods, demonstrating its generality for controlled preparation of complex heterostructures.

Instead of physical masking, the surface chemistry of the hard template can be engineered to control nucleation density and growth location. Zhao et al. [72] synthesized cauliflower‐like magnetic mesoporous silica microspheres by controlling the co‐assembly of CTAB and silica precursors on resorcinol‐formaldehyde (RF)‐coated magnetite particles. The process was driven by hydrophobic anchoring of CTAB to the RF surface, followed by electrostatic interaction with anionic silica oligomers. By adjusting the ammonia concentration, the kinetics of silica nucleation at RF surface were controlled, producing a unique cauliflower‐like structure with tunable surface roughness. This morphology enhanced surface hydrophobicity and promoted interactions with cell membranes. The versatility of this approach was further demonstrated in the synthesis of complex architectures like Fe_3_O_4_@SiO_2_@RF&PMOs multipods by Zhao et al. [73], in which the nucleation number of PMOs could be precisely controlled via mediating the surface properties of RF. Introducing electron‐withdrawing groups (e.g., ‐NO_2_) onto the RF layer enhanced surface nucleophilicity, lowered the critical nucleation concentration, and promoted multi‐site nucleation for multipod formation (Figure 6B). In contrast, PVP coating of the RF layer with weaker nucleophilic carbonyl groups raised the nucleation barrier and confined silica growth to a Janus morphology. This strategy was successfully extended to diverse nanomaterials, including Fe_2_O_3_ spindles, Fe_2_O_3_ cubes, highlighting its generality in topological engineering.

Furthermore, hard template can function as an intermediate interfacial layer that protects the core from degradation and initiates the growth of outer shell. Shi et al. [74] engineered a core‐shell structure by first coating hematite with a dense silica layer as hard template through hydrogen bonding‐mediated condensation. This layer protected the core from acidic leaching and provided an anionic interface for CTAB‐assisted electrostatic assembly of a mesoporous silica shell. The hematite core was then reduced under a controlled atmosphere to obtain magnetic nanoparticles (Figure 6C).

### 2.4 Crystallographic Anisotropy‐Guided Assembly

**FIGURE 7:**
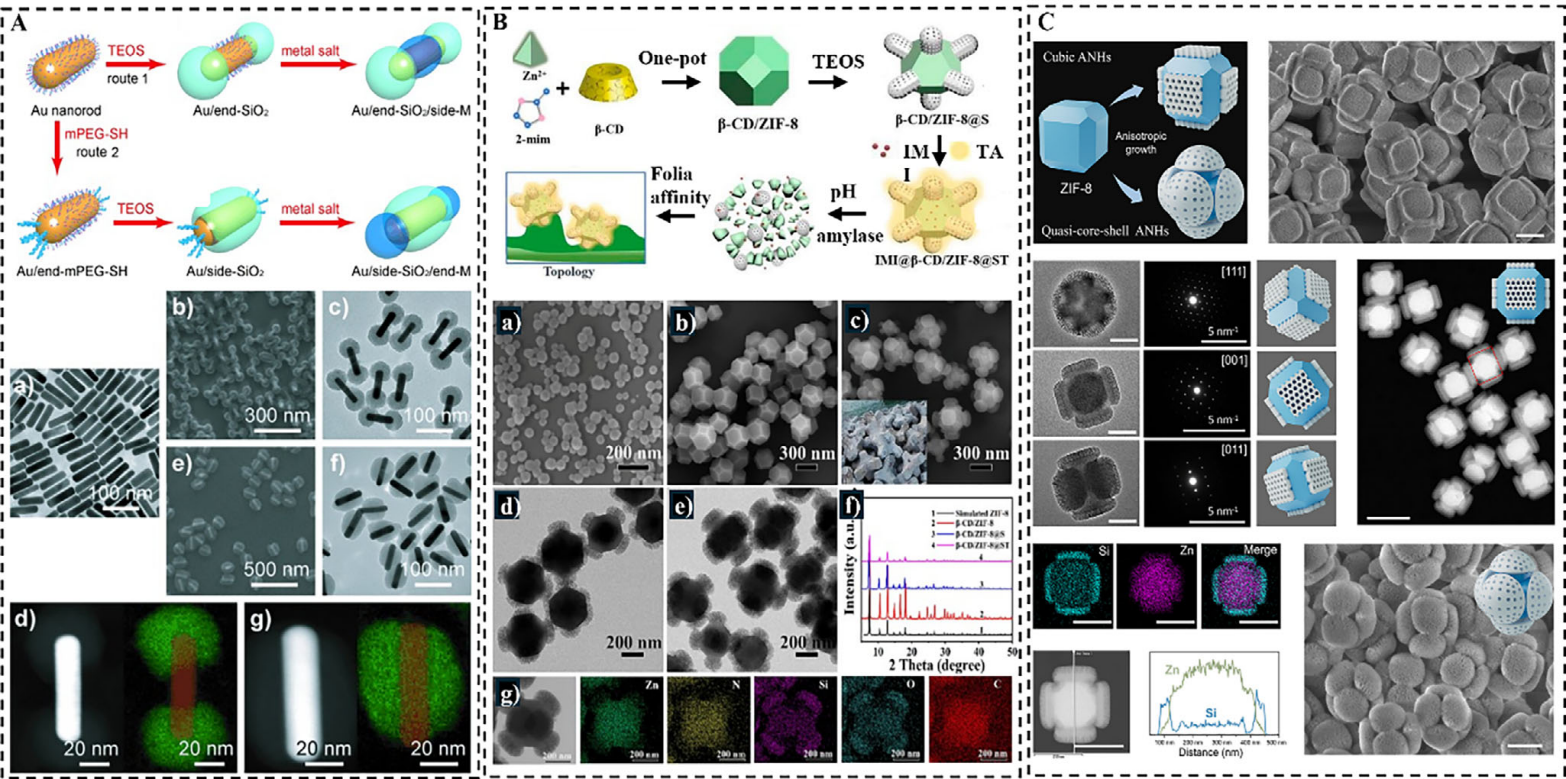
MOF‐directed site‐selective growth of silica: (A) Site‐selective growth of SiO_2_ on Au nanorod via anisotropic adsorption of CTAB. Reproduced with permission [75]. Copyright 2013, Wiley. (B) Accropode‐like ZIF‐8&mSiO_2_ mediated by anisotropic surface chemistry of β‑CD modified ZIF‐8. Reproduced with permission [78]. Copyright 2024, Elsevier. (C) Cubic and quasi‐core‐shell structured ZIF‐8&mSiO_2_ via dynamic interfacial equilibrium mediated by specific facets of ZIF‐8. Reproduced with permission [79]. Copyright 2025, WILEY.

Moving beyond introducing template as an external structural directing agent, this approach exploits the intrinsic crystallographic anisotropy of metal‐based materials to achieve site‑specific hybrid growth with programmable morphologies. A typical example is the noble metal nanocrystals like gold nanorods. Their intrinsic anisotropy results in different surface energies between high‐energy {111} tips and side facets, which dictates the facet‐dependent adsorption behavior of surfactant template like CTAB. On the high‐energy {111} tips, CTAB forms a less ordered and more loosely packed layer, which exposes the cationic headgroups and creates a higher effective positive charge density at the tips. Wang et al. [75] demonstrated this inherent selectivity by using a strong‐binding thiol‐terminated poly(ethylene glycol) ligand to selectively displace CTAB from the tips. This intervention suppressed silica nucleation at the ends of the nanorod, redirecting growth exclusively to the CTAB‐stabilized side facets (Figure 7A).

This strategy can be further extended to MOFs, where crystallographic anisotropy originates from distinct surface chemistries across different crystal planes, including metal‐coordination saturation, ligand termination, and their spatial arrangement [76, 77]. A representative example is ZIF‐8, whose {110} facets possess a higher zinc coordination density compared to the {100} facets, leaving the latter enriched with undercoordinated Zn^2+^ sites. Wang et al. [78] harnessed this anisotropy to achieve selectively growth of mesoporous silica “nanopods” exclusively on the {100} facets of ZIF‐8. This was enabled by pre‐modifying the ZIF‑8 surface with β‑cyclodextrin (β‑CD), which tuned surface hydrophilicity and morphology. The subsequent silica deposition was mediated by coordination between surface silanol groups and the hydroxyl groups of β‑CD, leading to the formation of octapod‑like heterostructures with enhanced adhesion and retention on biological surfaces (Figure 7B). This facet‐specificity was confirmed by using β‑CD&ZIF‑8 crystals with different {110}/{100} exposure ratios, which demonstrated that silica nucleation occurred exclusively on the {100} facets.

Instead of static selectivity, Li et al. [79] revealed a dynamic interfacial equilibrium governing the interaction between silica precursors and specific ZIF‐8 facets. They found that the hydrolyzed silicate species simultaneously stabilized the undercoordinated metal sites on {100} facets via coordination interaction between silanol groups and Zn^2+^ ion while chemically etching the {110} facets. By tuning this competitive interfacial process, they achieved precise morphological control, transforming pristine ZIF‐8&mSiO_2_ nanocubes into hexapod structures (Figure 7C). Furthermore, the above exposed crystalline surface of ZIF‐8 nanohybrids could induce secondary epitaxial growth of homo‐ or hetero‐MOFs, leading to the formation of nested or octopod‐shaped ZIF‐8&mSiO_2_&MOF nanohybrid. This work underscored the transition from simple facet‐selective growth to controllable morphological transformation via interfacial chemical reactions.

## 3 Applications in Antimicrobial Therapeutics

The above interfacial engineering strategies provide a versatile toolbox for constructing silica&metal‐based nanohybrids with tailored architectures, which can act as active functional modulators for antimicrobial therapeutics. This section establishes a direct correlation between heterostructures, physicochemical properties and their biological performance in four primary antimicrobial applications: (1) enhanced membrane disruption through physical and/or chemical attack; (2) spatiotemporally precise controlled therapy via stimuli‐responsive activation; (3) deep penetration and eradication of resilient biofilms through passive delivery or actively propelled mechanisms and (4) active targeting to bacteria via receptor‐mediated surface functionalization.

### 3.1 Membrane Disruption

Membrane disruption, achieved through physical, chemical or synergistic pathways, constitutes a fundamental antimicrobial mechanism of nanomaterials. Physical disruption typically employs nanostructures with well‐defined surface topographies, such as spiky or virus‐like morphologies, to impose direct mechanical damage on bacterial membranes via nano‐bio interactions. In contrast, chemical disruption relies on the release of antibacterial metal ions, such as Ag^+^ or Cu^2+^ to electrostatically interact with the negatively charged cell wall, disrupt membrane permeability and induce lethal oxidative stress.

**FIGURE 8:**
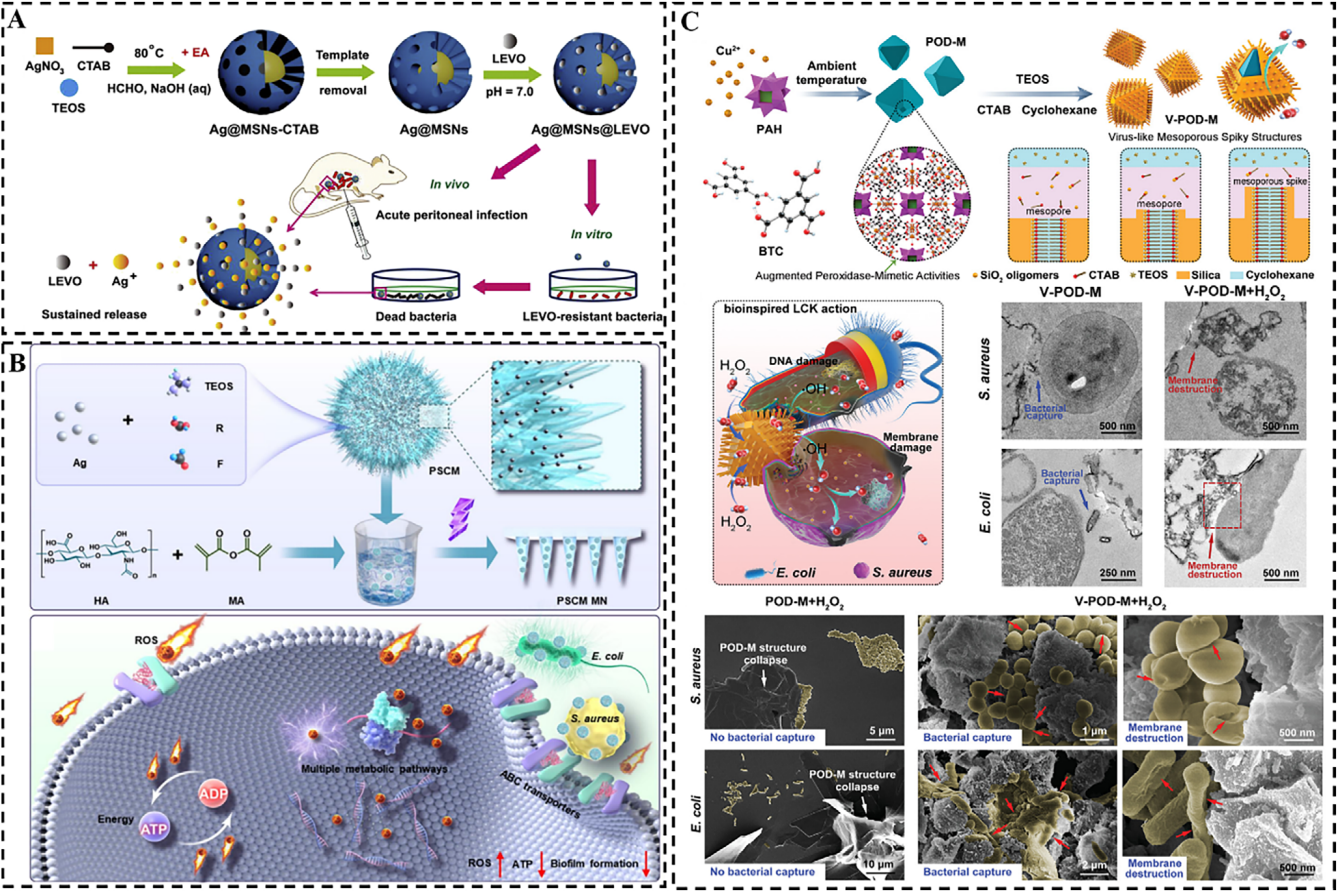
Bacterial membrane disruption: (A) Core‐shell type of Ag@MSN loaded with antibiotics for chemical disruption of the cell membrane. Reproduced with permission [80]. Copyright 2016, Elsevier. (B) Pollen‐like Ag@MSN core‐shell microsphere combined physical and chemical strategy for membrane disruption. Reproduced with permission [82]. Copyright 2025, Elsevier. (C) Spiky Cu‐based MOF@SiO_2_ with catalytic property to damage the cell membrane. Reproduced with permission [83]. Copyright 2021, WILEY.

A foundational chemical strategy employs rationally designed nanoparticle architectures, such as core‐shell or anisotropic structures as advanced nanocarriers to carry multiple antimicrobials and regulate their release kinetics. For instance, Gu et al. [80] engineered a core‐shell nanovesicle with a solid silver core encapsulated within an antibiotic‐loaded mesoporous silica shell. This architecture enabled the co‐delivery of two agents: the silica shell provided controlled release of antibiotics, while the silver core served as a sustained source of Ag^+^ ions, producing a synergistic effect against drug‐resistant infections (Figure 8A). Advancing this concept, Dong et al. [81] developed Janus “nano‐bullets” which were composed of a silver head and a mesoporous silica body with pre‐loaded with antibacterial cationic surfactants (CTAB). This compartmentalized design allowed for a cooperative release: sustained Ag^+^ ion released from the silver head alongside CTAB released from the silica body. The above nanohybrid was further functionalized with amino groups to enhance electrostatic targeting of bacterial cells, which concentrated the dual chemical attack at the membrane interface, leading to potent bactericidal activity.

Beyond chemical dominated membrane damage, a more advanced strategy integrates physical and chemical antibacterial mechanisms within a single nanocomposite to achieve synergistic effects. For example, Zhao et al. [73] fabricated mesoporous multipods composed of a central Fe_3_O_4_@SiO_2_@RF core surrounded by periodic mesoporous organosilica (PMO) nanocubes. This unique multipod topology enhanced bacteria adhesion via Van der Waals interaction, while the high surface area of the PMO component allowed for antibiotic loading. The magnetic cores further enabled effective bacterial segregation under an applied magnetic field, enhancing the nano‐bio interaction localized around the bacterial membrane. This integrated multicomponent nanoplatform demonstrated how morphology could enhance both physical capture and chemical delivery to inhibit bacterial proliferation. Similarly, Gao et al. [82] developed pollen‐inspired Ag@SiO_2_ particles with tunable spiky shells. They established a direct correlation between increased spike density and enhanced inhibition of *S. aureus* and Escherichia coli. The sharp protrusions physically disrupted membrane integrity, inducing oxidative stress and intracellular leakage, while the embedded silver nanoparticles provided sustained release of Ag^+^ ions, resulting in a powerful physico‐chemical synergy (Figure 8B).

Nanostructures can be engineered with catalytic activity to generate lethal chemical species in situ, integrating structural intervention with localized bactericidal action. Qiu et al. [83] synthesized a virus‐like peroxidase‐mimic (V‐POD‐M) that comprised a spiky silica shell for bacterial capture and a Cu‐based MOF‐derived core for catalytic sterilization. The spiky shell facilitated strong interaction and localized capture of bacteria. Meanwhile, the Cu‐based MOF‐derived core, containing Cu (II) and MoO_3_ catalytic centers, exhibited enhanced Fenton‐like catalytic activity, boosting the generation of highly cytotoxic •OH radicals (Figure 8C). This integrated “capture‐and‐kill” design, mimicking bacteriophage mechanisms, endowing the V‐POD‐M with rapid bacterial capture and near‐100% killing efficiency at low concentrations.

### 3.2 Stimuli‐Responsive Antimicrobial Nanoplatforms

Stimuli‐responsive nanoplatforms represent a pivotal advancement in precision antimicrobial therapy. These systems are engineered to remain biologically inert under physiological conditions, activating only upon exposure to specific pathological cues, such as the acidic pH or elevated glutathione (GSH), bacterial enzymes or external stimuli including ultrasound, magnetic field, and near‑infrared (NIR) light. This spatiotemporal control minimizes off‑target effect, enhances local bioavailability, and maximizes therapeutic efficacy. Considering the structural and compositional versatility of silica&metal‐based nanohybrid, diverse stimulus‑sensitive components, including pH‑labile coordination bonds, redox‑cleavable disulfide linkages, and plasmonic or magnetic metal‑based units, can be integrated into a unified architecture. Below, we examine endogenous and exogenous stimuli‑responsive systems and their mechanisms.

#### 3.2.1 Endogenous Stimulation

##### 3.2.1.1 PH Responsive Nanoplatform

**FIGURE 9:**
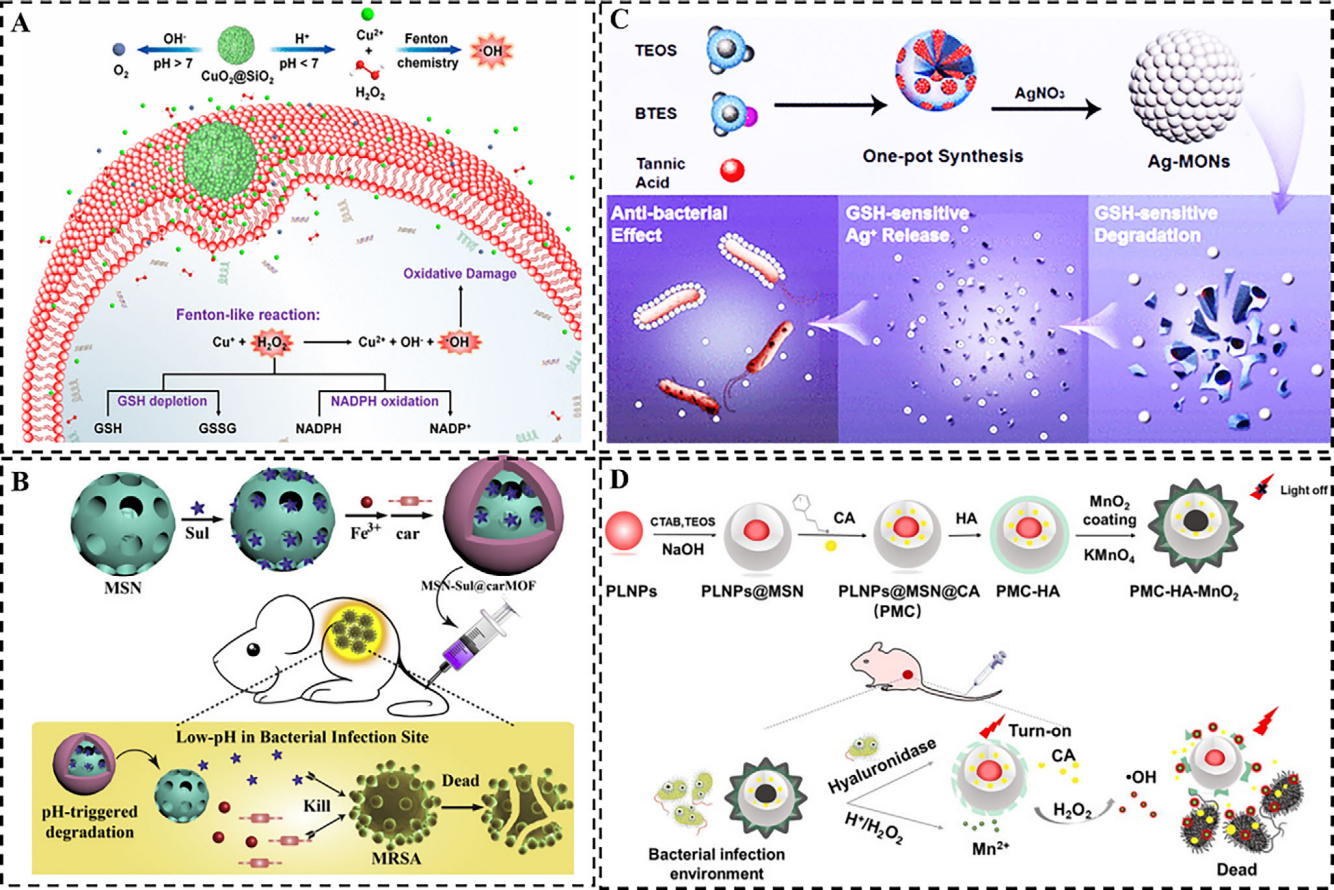
Endogenous stimuli‐responsive antibacterial nanohybrid: (A) pH‐responsive pomegranate‐like CuO_2_@SiO_2_ nanospheres. Reproduced with permission [86]. Copyright 2021, ACS Publications. (B) pH‐responsive carMOF@MSN. Reproduced with permission [87]. Copyright 2017, Elsevier. (C) GSH‐responsive Ag‐doped mesoporous organosilica nanoparticles. Reproduced with permission [90]. Copyright 2020, ACS Publications. (D) Enzyme‐responsive luminescence nanoparticles coated with MSN and capped with hyaluronic acid and MnO_2_. Reproduced with permission [94]. Copyright 2022, Elsevier.

Compared to healthy tissue with pH value of 7.4, the pH at the bacterial infection site is typically 4.5–6.6 due to the accumulation of bacterial metabolites and the associated host inflammatory response [84]. This distinct acidic microenvironment at bacterial infection sites offers a reliable endogenous stimulus for targeted therapeutic strategies. For instance, Chen et al. [85] employed a polydopamine (PDA)‐coated MSN for the in situ formation of silver nanoparticles and subsequent loading with curcumin (CCM). The release of both Ag^+^ ions and CCM was triggered by acidic pH and/or elevated ROS, governed by the protonation of amine groups and oxidative cleavage of hydrogen bonds within the PDA matrix. This intelligent co‐release strategy led to prolonged antibacterial activity. Similarly, Zhu et al. [86] developed pomegranate‐like CuO_2_@SiO_2_ nanospheres that decomposed under acidic conditions to initiate Fenton‐like reactions, generating lethal •OH for enhanced antimicrobial efficacy (Figure 9A). Zhang et al. [87] employed antibiotic‐loaded MSNs coated with a carbenicillin‐based metal‐organic framework (carMOF) shell. This MOF shell acted as a pH‐responsive gatekeeper, disintegrating under acidic environment to co‐release a β‐lactam antibiotic and a β‐lactamase inhibitor, thereby effectively eradicating antibiotic‐resistant bacterial strains (Figure 9B).

##### 3.2.1.2 GSH Responsive Nanoplatform

Glutathione (GSH), the predominant cellular antioxidant, neutralizes reactive oxygen species at infection sites through its reactive thiol group, thereby preserving intracellular redox homeostasis [88]. Its levels are characteristically elevated in bacterial infection microenvironments because of high oxidative stress [89]. This distinct biochemical signature can be used to trigger redox‐responsive drug release, clearing bacterial infections. Shao et al. [90] synthesized biodegradable mesoporous organosilica nanoparticles decorated with nanosized Ag using tannic acid (TA) as a structure‐directing and reducing agent. The embedded TA not only protected the Ag nanoparticles from oxidation but also enabled a GSH‐responsive degradation of the organosilica matrix. This degradation controlled the release behavior of silver ions, leading to a more potent and targeted antibacterial effect (Figure 9C).

##### 3.2.1.3 Enzyme Responsive Nanoplatform

Proliferating bacteria secrete a range of enzymes that create a unique biochemical microenvironment at the infection site [91]. These enzymes, which act as catalysts for biological and cellular reactions, not only influence tissue conditions and bacterial pathogenicity but also contribute to biofilm formation and resistance mechanisms [92]. This enzymatic activity can be harnessed as a precise trigger for designing enzyme‐responsive drug delivery systems, enabling controlled therapeutic release and enhanced antibacterial efficacy [93]. Yan et al. [94] coated mesoporous silica onto persistent luminescence nanoparticles. The mesoporous silica shell was loaded with cinnamaldehyde (CA) as antibacterial agent, sealed with a hyaluronic acid (HA) cap, and further coated with an MnO_2_ shell. In the presence of bacterial hyaluronidase and H_2_O_2_, the HA cap was degraded to release inner CA, while the MnO_2_ shell decomposed to activate luminescence for imaging, initiating chemodynamic therapy via a Fenton‐like reaction (Figure 9D).

#### 3.2.2 Exogenous Stimulation

Beyond endogenous triggers, externally applied physical stimuli, such as ultrasound, magnetic field, and light, offer unparalleled spatiotemporal precision. These triggers can be remotely controlled to initiate on‐demand antimicrobial action, minimizing off‐target effects while enabling deeper tissue penetration.

##### 3.2.2.1 Ultrasound‐Triggered Stimulation

**FIGURE 10:**
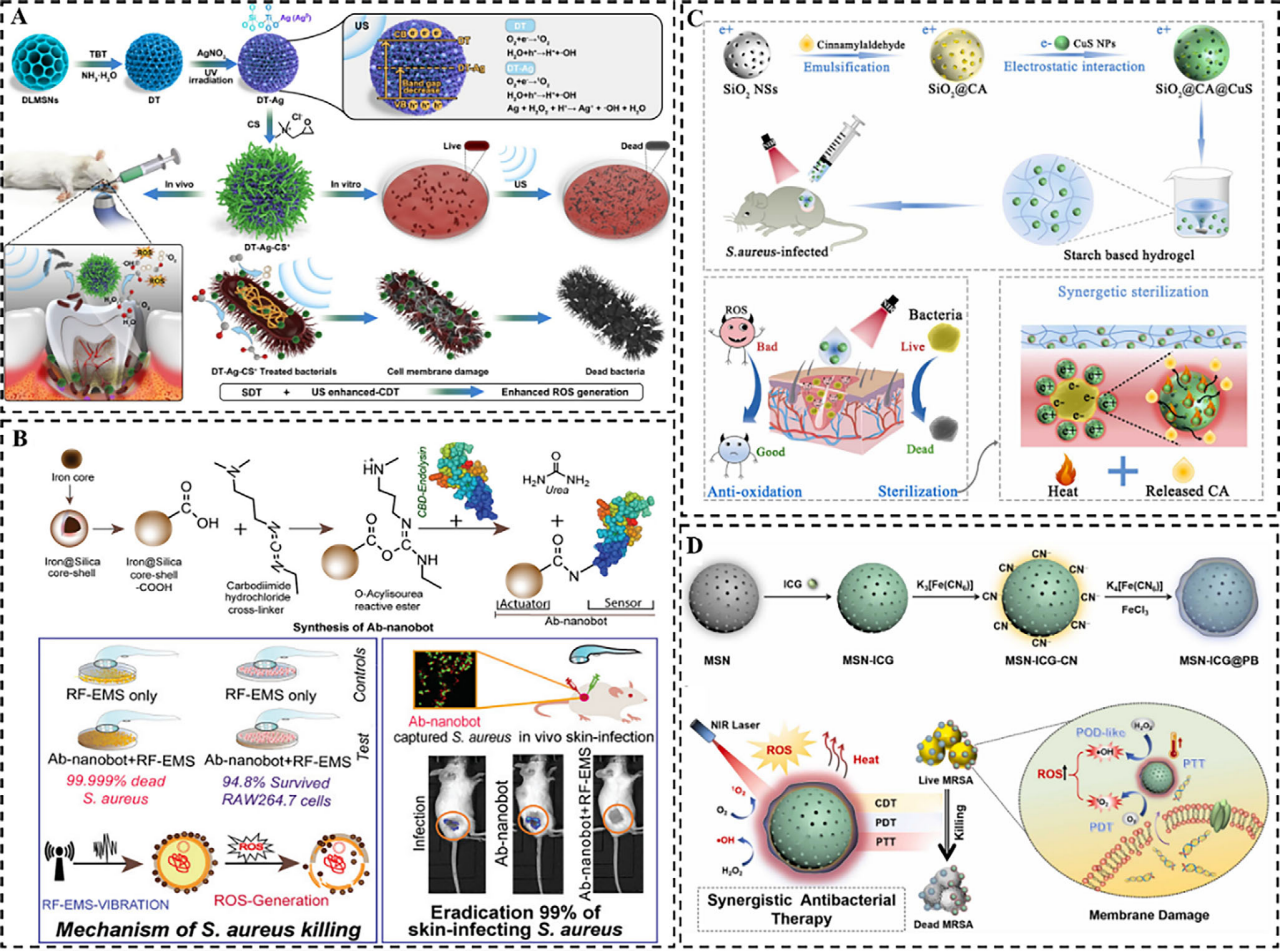
Exogenous stimuli‐responsive antibacterial nanohybrid: (A) Ultrasound‐responsive TiO_2_@SiO_2_ doped with Ag nanoparticle. Reproduced with permission [98]. Copyright 2023, Elsevier. (B) Magnetic‐responsive iron oxide@SiO_2_ modified with bacterial targeting endolysin. Reproduced with permission [101]. Copyright 2021, WILEY. (C) PTT‐responsive CuS@SiO_2_ doped with antibacterial reagent. Reproduced with permission [111]. Copyright 2022, Elsevier. (D) NIR‐responsive ICG doped MSN coated with PB for PTT/PDT/CDT synergetic therapy. Reproduced with permission [118]. Copyright 2024, Elsevier.

Ultrasound represents an ideal external stimulus for biomedical applications due to its noninvasive nature, deep tissue penetration, and cost‐effectiveness [95]. In antibacterial sonodynamic therapy, ultrasound activates sonosensitizers to generate reactive oxygen species, thereby inducing oxidative damage to bacterial cells. Furthermore, ultrasound‐induced cavitation enhances membrane permeability, which facilitates the penetration of therapeutic agents into deep‐seated infections and biofilms, improving treatment efficacy [96, 97]. Yang et al. [98] engineered a novel nano‐sonosensitizer comprising DMSNs‐supported TiO_2_ shell with deposited Ag nanoparticles. The DMSNs facilitated the growth of TiO_2_ and Ag and enhanced sonodynamic efficiency by lowering the acoustic cavitation threshold. The Ag coating played a dual role: its surface plasmon resonance effect facilitated the separation of electron‐hole pairs in TiO_2_ under ultrasound for enhanced ROS generation; while its Fenton‐like activity converted endogenous H_2_O_2_ into •OH, achieving a potent synergy between sonodynamic and chemodynamic therapy (Figure 10A).

##### 3.2.2.2 Magnetic‐Field Stimulation

Magnetism exhibits tissue penetration capabilities comparable to those of ultrasound. In magnetically guided antibacterial strategies, alternating magnetic fields (AMF) are employed to efficiently convert electromagnetic energy into thermal energy via superparamagnetic agents. This process enables targeted thermal ablation of bacterial cells while minimizing collateral damage to surrounding healthy tissues [99, 100]. Kim et al. [101] developed an antibacterial nanorobot by covalently conjugating a *S. aureus* targeted endolysin onto a magnetic iron oxide@SiO_2_ core‐shell nanoparticle. The nanorobots could detect and accumulate on the cell surface of *S. aureus*. Upon exposure to radiofrequency electromagnetic stimulation, the nanoparticles generated localized heat and ROS, leading to efficient bacterial cell death (Figure 10B). In a controlled release strategy, Vallet‐Regi et al. [102] decorated MSN with superparamagnetic iron oxide nanoparticles (SPIONs) and sealed the pores with thermo‐responsive polymer. Under AMF, the SPIONs produced localized heat, which induced a conformational change of the polymer gatekeeper, thereby uncapping the pores and triggering on‑demand release of encapsulated antibiotics. This design ensured direct heating at the biofilm interface, effectively combining magnetic hyperthermia with targeted drug delivery for biofilm disruption.

##### 3.2.2.3 Near‐Infrared (NIR)‐Light Stimulation

NIR light within the biological window (700–1000 nm) provides deep tissue penetration with minimal photocatalytic toxicity, making it ideal for remotely activating multifunctional nanocomposites [103, 104]. In this context, the nanohybrid of silica&metal‐based materials serve as versatile photoresponsive platform that allows for the integration of multiple functional components for efficient NIR‐driven antibacterial action. These systems operate primarily through two mechanisms: photothermal therapy (PTT), which converts NIR light to localized heat, inducing bacterial death via protein denaturation and membrane disruption; and photodynamic therapy (PDT), which harnesses NIR‑activated photosensitizers to generate cytotoxic ROS, often in synergy with light‑triggered drug‑release capabilities [105, 106].

##### 3.2.2.4 Photothermal Therapy (PTT)

Plasmonic nanoparticles (e.g., Au, Ag, CuS) could be used as effective NIR absorbers [107, 108]. Xu et al. [109] and Dong et al. [110] developed mesoporous silica coated Au‐Ag nanocages and Ag‐Bi nanoparticle co‐loaded mesoporous silica, respectively, as NIR activated photothermal agent. In both nano‐delivery platforms, localized photothermal heating directly damaged bacteria membranes under NIR irradiation while simultaneously accelerating the release of antibacterial Ag^+^ ions to achieve a synergistic photothermal‐chemo effect. This photothermal‐triggered strategy is further employed to controlled drug delivery. Wang et al. [111] co‐loaded copper sulfide (CuS) and a natural antibacterial active component (cinnamaldehyde, CA) into MSNs. Under NIR light, photothermal heat generated from CuS triggered the release of CA, enabling combined thermal and chemical ablation of bacteria (Figure 10C). Similarly, Vallet‐Regí et al. [112] coated triangular CuS nanoplates within antibiotic drug (Levo or Rif) loaded mesoporous silica shells. NIR irradiation induced photothermal heat from CuS, which triggered the co‐release of antibacterial Cu^2+^ ions and the encapsulated antibiotic drug, integrating thermal ablation with ion‐mediated toxicity and antibiotic action.

##### 3.2.2.5 Photodynamic Therapy (PDT)

Beyond PTT, PDT employs photosensitizers to generate cytotoxic ROS, eradicating drug‐resistant bacteria through a cascade of ROS‐mediated effects including immediate membrane damage, oxidation of key cellular components (e.g., lipids and proteins), and consequent cell death [113, 114, 115]. For instance, Su et al. [116] fabricated a core‐shell nanostructure by coating gold nanorods with mesoporous silica shell, which further loaded with curcumin as photosensitizer. This design harnessed the photothermal conversion capability of gold nanorod and ROS‑generating capacity of curcumin under light irradiation, achieving a synergistic effect of PTT and PDT. To further enhance bactericidal synergy, Chen et al. [117] designed an intelligent nanoplatform by coating lanthanide‐doped upconversion nanoparticles (UCNPs) with a hierarchical shell composed of dense silica and dendritic mesoporous silica. This structure enabled effective loading of methylene blue (MB) as photosensitizer and macromolecular lysozyme (LYZ). A bacterial hyaluronidase‐responsive valve was further modified on the particle surface to realize stimuli‐responsive release of LYZ. In this intelligent nanoplatform, the UCNP core converted NIR light to visible light, activating MB for ROS generation while the released LYZ degraded bacterial cell wall and exposed the bacteria to ROS. This bacteria‐responsive LYZ release and synergistic PDT effect achieved significant improved bactericidal efficiency, and prolonged antibacterial activity. Advancing the principle of multimodal synergistic therapy, Tu et al. [118] synthesized a core‐shell nanohybrid by loading indocyanine green (ICG) as photosensitizer into MSNs, followed by coating with a Prussian blue (PB) shell. Under NIR light irradiation, ICG simultaneously mediated PDT by singlet oxygen (^1^O_2_) generation and PTT through photothermal conversion. Meanwhile, PB shell catalyzed exogenous H_2_O_2_ at the infection site to produce highly toxic hydroxyl radicals (•OH), enabling CDT. Importantly, the localized heat generated from PTT enhanced bacterial membrane permeability, which facilitated the intracellular influx of ROS (^1^O_2_ and •OH) and further amplified thermal sensitivity. This cascade established a self‑reinforcing therapeutic cycle that integrates PTT, PDT, and CDT for efficient and potent bacterial eradication (Figure 10D).

### 3.3 Biofilm Penetration and Eradication

Microbial biofilms, protected by an extracellular polymeric substance (EPS) matrix, constitute a major therapeutic barrier due to their combined physical obstruction and chemical tolerance [119]. This EPS, composed of polysaccharides, proteins, and extracellular DNA, severely impedes the penetration of antibiotics, drastically reduces drug concentration at the bacterial surface, and fosters a protected microenvironment that promotes chronic, drug‐tolerant infections [120, 121].

**FIGURE 11:**
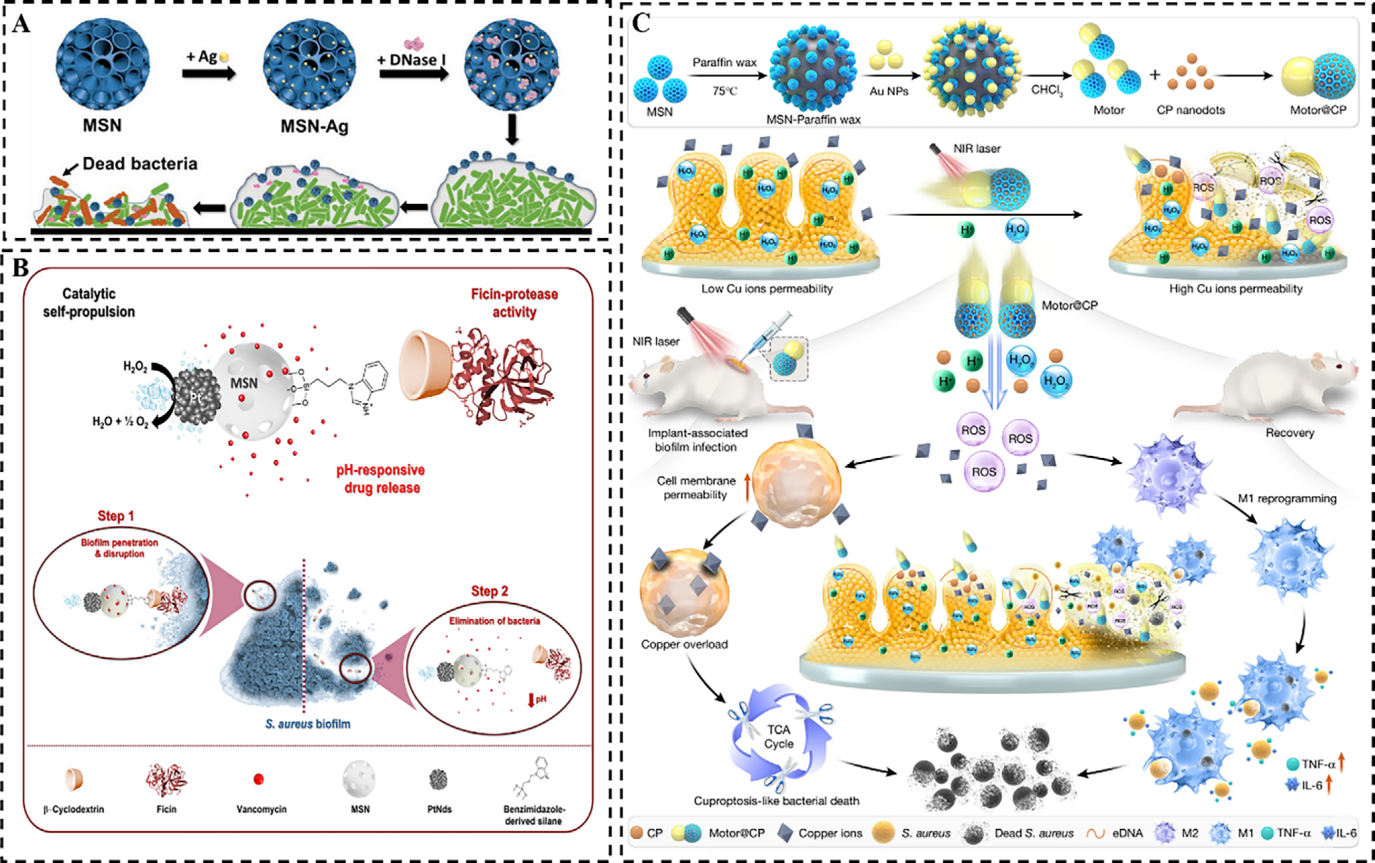
Disruption of biofilm formation: (A) Enzymatic degradation of biofilm via Ag@MSN loaded with DNase I. Reproduced with permission [123]. Copyright 2020, The Royal Society of Chemistry. (B) H_2_O_2_‐powered Janus Pt@MSN nanomotor for site‐selective releasing antibiotic to degrade biofilm. Reproduced with permission [126]. Copyright 2023, ACS Publications. (C) NIR‐powered Janus Au@silica nanomotor encapsulated within copper peroxide nanodots for biofilm eradication. Reproduced with permission [128]. Copyright 2022, Elsevier.

A primary strategy involves the integration of enzymatic, catalytic activity or physical disruption to directly degrade key components of biofilm [122]. For instance, Xu et al. [123] co‐loaded silver nanoparticles and the enzyme DNase I into large‐pore MSNs. The released DNase I hydrolyzed extracellular DNA within the EPS, weakening the biofilm integrity and facilitating the subsequent penetration of Ag^+^ ions. This process inactivated essential bacterial enzymes and induced oxidative stress, demonstrating a sequential synergistic attack (Figure 11A). Qu et al. [124] constructed mesoporous silica‐supported gold nanoparticles with intrinsic peroxidase and oxidase activities. These nanozymes generated a burst of ROS and promoted the conversion of H_2_O_2_ into highly oxidative •OH radicals, effectively degrading essential biofilm components to disrupt pre‐formed biofilms and inhibit new biofilm formation.

Self‐propelled nanomotors powered by internal or external stimulation represent another strategy for precise biofilm penetration and eradication [125]. Martínez‐Máñez et al. [126] designed a Janus platinum‐mesoporous silica nanomotor that was composed of platinum nanodendrites and an enzyme‐capped, drug‐loaded MSN. Upon reaching the biofilm via H_2_O_2_‐induced self‐propelled motion, the enzyme was released to hydrolyze the proteinaceous components of the EPS while the acidic biofilm microenvironment triggered pH‑responsive drug release, achieving synergic antimicrobial action (Figure 11B). In a light‐powered system, Li et al. [127] engineered a Janus nanomotor by asymmetrically coating copper peroxide nanodots‐loaded MSN with gold nanoparticles. Under NIR irradiation, the nanohybrid underwent self‐thermophoretic propulsion, actively penetrating into deep biofilm regions. The acidic microenvironment then triggered the decomposition of copper peroxide, initiating a Fenton‐like reaction that generated •OH radicals. These radicals chemically disrupted the EPS while enhancing the intracellular uptake of copper ions, triggering a cuproptosis‐like pathway for bacterial eradication. In another study, Wang et al. [128] coated photothermal gold nanorod core with a mesoporous silica shell, which was co‐loaded with a photosensitizer (indocyanine green, ICG) and a nitric oxide (NO) donor. Under NIR light, the gold core generated localized heat for PTT and triggered the release of NO, inducing oxidative/nitrosative stress. Simultaneously, ICG produced ROS for PDT. This tri‑modal attack combined physical disruption via heat with comprehensive chemical damage through ROS and NO, resulting in potent biofilm eradication (Figure 11C).

### 3.4 Active Targeting to Pathogen Bacteria

While conventional antibacterial nanoplatforms primarily rely on passive accumulation or stimuli‐responsive release triggered by internal or external factors (as mentioned in Chapter 3.2.), these strategies often lack selectivity toward pathogenic bacteria. This non‐specificity poses a potential risk as it can inadvertently disrupt beneficial microbial communities, which play an essential role in human health, and may predispose patients to secondary infections [129, 130]. To address this challenge, recent efforts have focused on designing smart nanoplatforms which can actively target bacteria colonized at the infected sites. Such active targeting strategies can minimize off‐target effect on healthy host cells, reduce the required dosage and frequency of nanomedicine administration [131].

Active targeting is typically achieved through surface functionalization with ligands that specifically recognize and bind to molecules or receptors overexpressed on bacteria cell walls. Commen targeting ligands include antibodies, antimicrobial peptides, aptamers, and carbohydrates [132, 133]. An emerging approach involves coating nanoparticles with bacterial membranes, which endow them with surface antigens for immune evasion and homing to homologous bacteria strains [134, 135]_._ Despite its potential, the application of active targeting strategies in silica&metal‐based materials is still in its infancy. In the following section, two representative ligand‐receptor based strategies are discussed as illustrative exmaples.

#### 3.4.1 Antigen‐Antibody Recognition‐Based Targeting

The antibodies high‐affinity ligands that can specifically bind to antigens presented on bacterial surfaces. For instance, Wang et al. [136] engineered a theranostic platform by coating Fe_3_O_4_ nanoparticle with a mesoporous silica shell. The antibotic vancomycin was loaded into the pore channel, which was then capped with sulfonated hyaluronic acid (S‐HA) to enable stimuli‐responsive release. The above nanosystem was finally functionalized with anti‐*S. aureus* antibody and immoblized on the suface of a magnetic glassy carbon electrode (MGCE). This design exploited the specific antigen‐antibody binding interaction for detecting the *S. aureus* in the whole blood. With increased amount of *S. aureus* captured on MGCE, the bacteria‐secreted hyaluronidase degraded the S‐HA cap, triggering the localized release of vancomycin for targeted bacteria killing.

#### 3.4.2 Peptides Recognition‐Based Targeting

Composed of short amino acid chains, peptides offer distinct advantages for targeted delivery, including high specificity, excellent biocompatibility, high safety profile, ease of chemical modification, and diverse natural or synthetic sources. Jayawardena et al. [137] developed a NIR‐responsive nanoplatform for antibacterial therapy. The nanoplatform was constructed by coating gold nanorods with an antibacterial drug‐loaded mesoporous silica shell, which was further encapsulated with a thermo‐sensitive liposome. The liposomal surface was functionalized with a mycobacteria‐targeting peptide, enabling active targeting onto the mycobacteria surface. Upon NIR irradiation, the gold nanorod generated localized heat, disrupting the liposome layer and triggering the on‐demand release of antibacterial drugs.

## 4 Conclusions and Perspectives

This review has systematically examined the pivotal role of interfacial chemistry as a central design principle to fabricate silica&metal‐based heterostructures for advanced antimicrobial applications. Moving beyond material synthetic methodologies, we have elucidated how precise interfacial engineering through electrostatic, coordinative, template‐assisted, and crystallographic anisotropy‐directed reactions, enables the integration of structurally and chemically disparate components into one nanoplatform. This chemical control allows deliberate manipulation over the architecture and functionality of the resulting hybrid systems.

The integration of silica plays two complementary roles. First, it acts as structural‑mediating matrix that mitigates the inherent limitations of metallic‐based materials (e.g., aggregation, structural instability, nonspecific cytotoxicity) and facilitates the construction of well‐defined nanoarchitectures such as encapsulated nanocomposites, core‐shell structures and anisotropic Janus particles. Second, silica‐based materials serve as active functional modulators that enhance bio‐interactions, enable controlled cargo loading and release, and facilitate surface functionalization to achieve tailored stimuli‐responsive behavior.

These interfacial‐engineered hybrids exhibit enhanced antibacterial efficacy in four major fields: (1) synergistic membrane disruption through physical and/or chemical mechanisms; (2) spatiotemporally controlled multimodal therapy enabled by stimuli‐responsive activation of endogenous or exogenous triggers; (3) effective biofilm penetration and eradication via enzymatic, catalytic, or multiple‐based strategies and (4) active targeting to bacteria via surface functionalized with receptor‐specific ligands. Although significant progress has been made in this field, critical challenges must be addressed to bridge the gap between laboratory innovation and clinical translation. Below, we outline key considerations and prospective directions:

1. Although electrostatic and coordinative bonds currently dominate interfacial strategies for constructing silica&metal‐based composite, future designs could exploit cooperative interactions such as hydrogen bonding, hydrophobic interactions, supramolecular recognition to assemble more complex and stable heterostructures. Furthermore, precise interfacial engineering (e.g., tuning bond stability, coordination‑site lability, and nanoscale diffusion pathways) can be employed to control the spatiotemporal kinetics of metal‑ion release. These strategies enable programmable antimicrobial profiles from rapid bactericidal bursts to sustained, long‐term release.
2. Coating pH‐ or temperature‐sensitive materials such as CuO/Cu_2_O or ZIF‐8 with silica remains challenging, due to the traditional alkaline conditions typically required for the hydrolysis of silica precursor (e.g., TEOS) and subsequent high‐temperature calcination to remove co‐assembled template (e.g., surfactant and polymer). This limitation becomes particularly relevant for emerging rough silica nanoparticles with tunable surface topographies, which offer superior bio‐interfacial properties. Future work should prioritize mild and protective coating methodologies such as using stabilizing templates or surface modifications to enable the integration of these sensitive metallic components with advanced silica nanostructures.
3. The integration of mesoporous silica with MOFs generates hierarchical pore architecture, but structure‐property relationships within these composites remain to be better understood. Precise control over pore geometry, size, and interconnectivity is crucial to maximize therapeutic loading capacity and to facilitate hybridization with a broad range of metal/metal oxides. Beyond structural engineering, it is essential to elucidate how nanoconfinement within these hierarchical pore systems modulates interfacial interactions, exposure and accessibility of active sites, and catalytic or antibacterial activity, enabling design of hierarchical nanohybrids with superior synergistic performance.
4. The current research mainly focuses on traditional antimicrobial agents such as Au, Ag, CuO, ZnO and Cu/Zn based MOF. The field should broaden its scope to include metallic elements with higher intrinsic antibacterial potency, lower cytotoxicity, and greener synthesis methods. Moreover, beyond conventional ion‐release mechanisms, future work should employ unique metallic properties to induce non‐conventional bacterial death pathways, such as ferroptosis or cuproptosis, thereby opening new therapeutic avenues.
5. Most anti‐biofilm studies employ single‐species models, which poorly reflect the clinical complexity of polymicrobial biofilms that exhibit enhanced antibiotic tolerance and pathogenicity. Future platforms could be designed and validated against these multi‐species communities to ensure clinical relevance and efficacy.
6. Current synthetic strategies for silica‐based hybrids incorporating metal oxides or MOFs often involve complex designs such as multi‐functional, multi‐interfaces, multi‐elements, multi‐chemistry, et al. While scientifically attractive and potentially effective, such nanoarchitectures are often irreproducible and unscalable for real‐world medical or clinical applications. Moreover, silica&MOF nanohybrids remain relatively expensive for antibacterial applications, despite ongoing efforts to address this limitation. To bridge the gap between laboratory innovation and practical application, the synthesis protocol should be controllable, cost‐effective, and simplified to enable scalable production.
7. Although silica nanoparticles are generally recognized as safe by the American Food and Drug Administration, the overall biosafety profile of silica&metal‐based materials is governed by multiple factors, such as surface modifications, size‐dependent behavior, metallic components and ligands especially for MOFs. Long term toxicity, chronic exposure effects, comprehensive biodistribution and toxicology studies must be rigorously evaluated through standardized methods. To minimize off‐target effects and mitigate long‐term toxicity, future designs of silica&metal‐based materials should integrate two complementary strategies: one is active targeting via surface functionalization with specific ligands such as antibodies, aptamers, antimicrobial peptide; the other is safety‐design strategy such as engineering renal‐clearable sizes, developing biodegradable silica hybrids, or designing magnetically retrievable system.

In conclusion, interfacial chemistry serves as an important design toolkit for integrating metallic‐based components with silica to construct versatile heterostructures with programmable antibacterial functions. A deeper understanding of structure‐property‐function relationships will be the key to developing next‐generation nanomaterials. By addressing the outlined challenges, such rationally engineered heterostructures hold significant promise for combating drug‐resistant infections and translating innovative nanomaterial design into effective clinical therapies.

## Conflicts of Interest

The authors declare no conflicts of interest.

## Data Availability

The authors have nothing to report.
